# Supplementary material for: Impacts of and survival adaptations to the COVID-19 pandemic among the hill tribe population of northern Thailand: A qualitative study
Source: PLoS One. 2021 Jun 4;16(6):e0252326. doi: 10.1371/journal.pone.0252326 (PMC8177523; doi:10.1371/journal.pone.0252326)
Supplement: S1 Appendix — (DOCX) [file pone.0252326.s001.docx]

**Questions guideline**

| **Item** | **Question** |
| --- | --- |
| **1.The impacts from the covid-19 epidemic** | a) How do you get impacts from the covid-19? |
|  | b) How do you have any health impact from the disease epidemic? |
|  | c) How do you have any economic impact from the disease epidemic? |
|  | d) How did you get the impact from the government control and prevent measures? |
|  | e) How did you have relationship problem from the distancing policy and measure? |
|  | f) How do you have the disparity of the support from government due to having the impact the disease |
|  | g) Regarding school attending of your children, how do you have impact form the problem? |
| **2.The information on the adaptation from the covid-19 epidemic** | a) How do you adapt in self-care to prevent the disease |
|  | b) How do you do to protect your family member? |
|  | c) How do you modify your daily practice in attending social activities? |
|  | d) Under the social distancing, how do you keep your relationship within family members? |
|  | e) How you maintain your family financial status? how do you a medical access in case of having a family member who needs to access a medical care regularly |
|  | f) How do adapt to the government control and prevent measures? |
|  | g) How your community apply to prevent and control the disease to the community members? |
