# Supplementary material for: Impacts of and survival adaptations to the COVID-19 pandemic among the hill tribe population of northern Thailand: A qualitative study
Source: PLoS One. 2021 Jun 4;16(6):e0252326. doi: 10.1371/journal.pone.0252326 (PMC8177523; doi:10.1371/journal.pone.0252326)
Supplement: S2 Appendix — (DOCX) [file pone.0252326.s002.docx]

1.ผู้สัมภาษณ์ 2.ผู้ถูกสัมภาษณ์

**P1 สุธิมา.m4a**

1. จะถามคุณแม่ว่าโควิดคุณแม่ได้รับผลกระทบมั้ยครับ ได้รับอย่างไรบ้างครับ

2. ก็ป้าเป็นคนเย็บผ้า แฟนซ่อมรถ อยู่ที่เดียวกัน มันก็หายไป ถ้าเป็นเรื่องซ่อมรถ เศรษฐกิจแย่ ลูกค้าก็ลดลง คนเย็บผ้า ถ้ามีกิจกรรมอะไร ก็ไม่มาสั่งตัด

1. คนหายไปอัตโนมัตเลยหรอครับ ที่หายไป

2. ไม่ขนาดนั้น แต่ก็ไม่ได้ทำเหมือนเดิม

1. จากเดินที่ออเดอร์เยอะเรื่อยๆ ตอนนี้ก็หายไป

2. ค่ะ

1. หายไปเยอะเลย แต่ถ้าอะไรนะครับ

2. แต่ป้าทำได้หลายอย่าง อย่างชุดไทย ชุดพื้นเมือง ก็ไม่ตกงาน ตอนนี้ไม่ว่าชุดของผู้ชายผู้หญิงนี้เย็บได้หมดเลย

1. อาชีพก็เหมือรับจ้างทั่วไปนะครับ ก็มีผลกระทบ แต่มีหลายอาชีพก็พอได้ใช่มั้ยครับ

2. ค่ะ

1. แล้วแฟนที่ซ่อมรถละครับ หรือว่าแค่ลดลง

2. แฟนก็ทำๆไป เหมือนแต่ก่อนมีวันละ 10 คน ตอนนี้บางวันก็เหมือนไม่ได้ทำสักอย่าง ไม่ได้ทำอะไรเลย

1. ค่าใช้จ่ายนี้เท่าเดิมมั้ยครับ

2.เท่าเดิมค่ะ

1.เราต้องกินอยู่ค่าโทรศัพท์นี้เท่าเดิมใช่มั้ยครับ

2.ค่ะเท่าเดิม

1.แต่รายได้ลดลง ตั้งแต่มีโควิดใช่มั้ยครับ แสดงว่าตั้งแต่ปี 2563 ที่โควิดเข้ามาเนี้ย รายได้ลดลงใช้มั้ยครับแล้ว

2.ค่ะ

1.แล้วหนี้สินละครับ เพิ่มขึ้นมั้ย

2.หนี้สินก็มีทุกปีกู้เงินกองทุนทุกปีค่ะ

1.รายได้ลดลงแต่รายจ่ายเท่าเดิมนะครับ

2.ค่ะ

1.แล้วอย่างนี้ทำให้หนี้สินเพิ่มขึ้นมั้ย หรือต้องไปกู้เพิ่มมั้ย

2.ไม่เพิ่มค่ะเท่าเดิม

1.ก็คืดประคองตัวได้อยู่นะครับ

2.ค่ะ

1.อันนี้ด้านเศรษฐกิจนะ โควิดที่ส่งผล อาชีพยังเปลี่ยนใช่มั้ยครับ ทำอย่างอื่นเสริม ลุงก็ยังซ่อมรถอยู่เหมือนเดิมนะครับ ยังไม่หันเหไป

2.ค่ะ ยังไม่เปลี่ยน

1.แล้วทางด้านสุขภาพละ จากโควิดที่ผ่านมามีผลกระทบต่อสุขภาพอะไรมั้ยครับ

2.ไม่มีค่ะ มีแต่โรคประจำตัวที่เป็นโรคซึมเศร้ากับกระเพราะค่ะ

1.เดี๋ยวก่อนนะครับ โรคซึมเศร้ากับโรคกระเพราะนี้เป็นมานานหรือยังครับ

2.เป็นมานานแล้วค่ะ เกิดมาก่อนโควิดเข้ามา

1.แล้วพอโควิดเข้ามาทำให้อาการซึมเศร้าเพิ่มมากขึ้นมั้ยครับ เศรษฐกิจกินไม่ดี เครียดหนักกว่าเดิมมั้ยครับ

2.ก็ไม่ค่อยเครียดเท่าไหร่ค่ะ เพราะว่าบ้านเราก็ไม่เคยมีค่ะ ป้าก็เป็น อสม นะ ก็เลยได้อบรมตลอด

1.เราก็เลยมีความรู้ รู้เท่าทัน

2.ค่ะ

1.ผลกระทบการโควดที่ส่งผลกับเรา ติดก็ไม่ได้ติด เป็นก็ไม่ได้เป็น แล้วด้านจิตใจมีมั้ยครับ เรื่องวิตกกังวล

2.ก็มีอยู่ค่ะ ก็คิดว่าจะเป็นแบบนี้ไปตลอดชีวิตหรือป่าว

1.ก็ประมาณว่าจากที่เราเป็นอยู่นี้นะ แบบว่าเดี๋ยวก็ระบาดอีกแล้ว เดี๋ยวก็มา

2.ค่ะ ก็คิดว่าจะเป็นแบบนี้ตลอดหรือป่าวค่ะ สถานการณ์การอยู่การกินจะเป็นแบบนี้ที่คิดที่กังวล

1.แล้ววิถีชีวิตเปลี่ยนไปเลยมั้ยครับ สมมติสมัยก่อนที่มีการไปมาหาสู่ การไปงานแต่งงานเลี้ยงพอโควิดมารู้สึกว่ามันกระทบมั้ย

2.กระทบค่ะ ก็ เป็นญาติก็ไม่ได้ไปหากัน มีลูกหลานที่กรุงเทพจะมาเยี่ยมก็ไม่ได้มา

1.แล้วเรื่องที่เขาให้เราอยู่ห่างกัน ร้านค้าปิด ตลาดปิด ใส่แมสนี้กระทบต่อเรามั้ยครับ การสแกนเข้าร้าน ล้างมือ

2.ยุ่งยากค่ะ ถ้าเราไป บางทีลืมแมสก็ต้องวิ่งไปเอา หรือซื้อหน้าสถานที่ ลืมหน้ากาก

1.ต้องปรับตัว ตอนนีเป็นปีหนึ่งละ ปรับตัวได้หรือยังครับ

2.ตอนนี้ก็เอาแมสใส่ไว้ในกระเป๋าบ้างใส่เบาะรถบ้าง ไว้หลายๆอันค่ะ ถ้าเราลืมก็คิดได้ก็จะได้หยิบ

1.วิธีการในการปรับตัว คิดว่าเราเริ่มปรับตัวได้หรือยังครับ เคยได้ยิเรื่องวิถีชีวิตใหม่มั้ยครับ

2.กินร้อนช้อนกลางก็ทำตั้งแต่ยังไม่มีโควิดค่ะ

1.ทำเพราะอะไรครับ

2.ก็ทำเพราะว่าการกินการอยู่มันเปลี่ยนไป โรคก็มาเรื่อยๆ โรคมาใหม่เยอะๆ ป้าก็เป็น อสม ก็เคยได้ยินหมอบอกค่ะ

1.แสดงว่าการที่เราเป็น อสม ก็เป็นโอกาสดีอย่างหนึ่งนะครับ เป็นยังไงบ้าง

2. ได้ความรู้ค่ะ ได้ช่วยชาวบ้าน ป้าเป็น อสม มา 20 ปีแล้วค่ะ แต่ก่อนถ้าไม่มีบัตรไทบเวลาไปหาหมอก็จะต้องเสียตังนะคะ ป้าก็ทำ แต่ก่อนก็อาศับรักษาฟรี เข้าเป็น อสม คุ้ม พ่อแม่ลูกสามี แต่ก่อนไม่ได้เงินเดือนสักบาท แต่งานก็ไม่ได้หนักขนาดนี้ แล้วก็เป็นมา อันนี้ในอำเภอแม่ฟ้าหลวงเราก็มีสมาชิกค่ะ ป้าก็แก่มา อาศัยอันนั้นบ้าง ตอนนี้ทั้งประเทศไทยก็มีกลุ่มชาปณกิจที่หักตังช่วยเหลือกลุ่ม อสม ได้ทุกคน บางคนไม่มีบัตรก็ถือว่าไม่ได้นะคะ

1.ตอนนี้ป้าได้บัตรไทยละเน๊าะ แล้วถ้าคนที่ไม่ได้บัตรไทยน้ำยังไงครับ

2.หัวหน้าอนามัยบอกว่าเข้าได้

1.ถ้ามีบัตรไทยนี้ได้อยู่แล้ว แต่ถ้าคนไม่มีละครับ

2.บางคนที่ไม่มีก็ไม่อยากได้ เพราะว่าเขามีลูกหลายคน เดี๋ยวลูกอิจฉากัน เดี๋ยวแบ่งกันไม่ลงตัวค่ะ

1.ป้าเป็น อสม ถือว่าเป็นโอกาสดีที่อยู่ใกล้หมออนามัย ในช่วงเกิดโควิดนี้ก็ถือว่าเป็นบุคคลสำคัญคนหนึ่งในชุมชนนะครับ อสม มาช่วงในการกักตัว ช่วงวัดไข้แล้วคนในหมู่บ้านธรรมดาละครับ ถ้าเป็ชาวบ้านธรรมดาที่ไม่ได้เป็น อสม เหมือนป้าละครับ

2.ไม่ค่อยรู้เรื่องค่ะ ชาวบ้านก็ร่วมไม้ร่วมมือ ไม่ค่อยรู้เรื่องก็ไม่กลัวค่ะ อย่างลูกหลายเขามาจากทำงานไกล เขาให้กักตัวค่ะ เขาไม่ฟังก็กินรวมกัน พอป้าเป็น อสม ไปบอกเขา เขาก็โกรธเรา โดนด่ามาอีก แต่เราก็กลัว ถ้าบ้านหลังนั้นมีคนมาจากที่ไกลไม่กักตัวแล้วคนบ้านเขาก็ไปข้างนอกอย่างเงี้ย

1.ก็มีเยอะใช่มั้ยครับ ช่วงปีที่ผ่านมา ไม่มีงานทำก็ติ้งกลับมาบ้าน ตามมาตรการก็ต้องกักตัว อยู่ห่างกัน คนถือว่าให้ความร่วมมือเยอะแค่ไหนครับ

2.ครึ่งต่อครึ่ง

1.ครึ่งหนึ่ง คนที่ให้ความร่วมมือก็มีอยู่ คนที่ให้ความร่วมมือดีก็มี

2.คนที่กลัวจริงๆก็มี อย่างป้าไปสำรวจลูกน้ำยุงลาย บางบ้านไม่ให้ป้าเข้าบ้านก็มี

1.ทั้งๆที่เป็นคนในหมู่บ้านนี้ใช่มั้ยครับ

2.ค่ะ บ้านเรามีหลายเผ่าค่ะ 9 เผ่าค่ะ ป้าเป็นไทยใหญ่ มี จีน ลั้ว ลีซอ ม้ง เมี่ยว คนไทย อาข่า มูเซอร์

1.9 เผ่านี้เขาแยกเป็นหมู่กันมั้ยครับ

2.ไม่ค่ะ อยู่สผมกัน แต่ศาสนาก็ไม่เหมือนกันนะ อิสลาม คริสต์ ผี พุทธ แต่เข้ากันได้ สมมติ ปีใหม่อาข่าเราก็ไม่ว่าอะไรเขา เขาก็ไม่ว่าอะไรเรา

1.ศาสนาส่วนใหญ่อะไรมาสุดครับ

2.พุทธ คริสต์ ผี อิสลามไม่เยอะ ส่วนมากเป็นคนจีนที่นับถืออิสลามค่ะ แต่ก่อนที่ไม่มีโควิดก็มีเข้ามาเยี่ยมกันค่ะ มีมัศยิด มีวัดของเขาค่ะ

1.ขอโทษนะครับ ผมคุยกับป้านานแล้วป้าชื่ออะไรนะครับ

2.ป้าชื่อสุธิมาค่ะ

1.ป้าพุทธหรือคริสต์ครับ แล้วอายุเท่าไหร่

2. พุทธค่ะ อายุ 50 ค่ะ แต่อายุจริงๆ 53 ค่ะ แต่ก่อนเราไม่ค่อยรู้เรื่อง

1.แล้วมาตรการที่เขาช่วยเหลือเกี่ยวกับโควิดละครับ รู้สึกว่าเราได้รับความช่วยเหลืออะไรเรามั้ยครับ

2.ช่วยค่ะ คนละครึ่งรอบที่ 1 รอบละ 5000 ลูกหลานสมัครให้เลยได้ค่ะ แต่หลังๆนี้ไม่ได้อะไร

1.โควิดมันมาเป็นปีเลยเน๊าะ แล้วคิดว่ามาตราการที่ได้รับคิดว่าพอใจหรือไม่พอใจอย่างไรบ้างครับ

2.ดีกว่าไม่ได้ค่ะ ได้มาก็ทำบุญบ้าง ไม่เอาทั้งหมด ถ้ามันเป็นของรัฐบาลไม่อยากให้เข้าแต่เราคนเดียวค่ะ เงิน อสม ก็เหมือนกันเข้าทีละเดือนป้าก็จะเอาไปบริจาคให้คนแก่หรือซื้อขนมไปให้ ป้าไม่เอาคนเดียว

1.ถ้าสมติเรียกร้องได้ อยากให้รัฐบาลช่วยอะไรมั้ยครับ

2.ไม่อยากขอร้อง ถ้าเขาให้มาก็เอา ถ้าเขาไม่ให้มาก็ชั่งเขาอย่างนั้นค่ะ

1.ทำไมละครับ

2.ก็เราไปเรียกร้องเราก็ไม่ค่อยรู้กฎหมาย ถ้าไปเรียกร้องสิทธิ์แบบนี้ค่ะ

1.ก็ไม่รู้จะเรียกร้องอะไร

2.ค่ะถ้าไปเรียกร้องแล้วผิดกฎหมาย เดี๋ยวเป็นเรื่องเป็นราว ถ้าเราไปเรียกร้องตำรวจให้เราผิด เราก็ต้องยอมผิดอย่างนี้ค่ะ ก็คิดแบบนั้น

1.รู้สึกว่าเขาเหนือกว่าเราหรอครับ เขามีอำนาจกว่าเราหรอครับ

2.ใช่ค่ะ ยังไงเราก็ไม่มีทางสู้เลยชั่งเขา

1.ทุกวันนี้ครอบครัวป้าเอง โรคโควิดที่เข้ามาแบบนี้ก็คืออยู่กับเรามาเป็นปีแล้วปีนี้ก็มาอีกแล้ว ก็คือยอมรับความเป็นจริงได้ อาจะเครียดบ้างได้รับผลกระทบบ้าง แต่ก็ยอมรับ เกิดแล้วก็ต้องเกิด ไม่ไปเรียกร้องอะไรเท่าไหร่

2.ใช่ค่ะ ถ้าเขาให้ก็เอา ไม่ให้ก็ชั่งเขา อยู่ด้วยลำแข้งตัวเองก่อนค่ะ

1.แล้วคนในหมู่บ้านละครับ คนที่คิดแบบป้านี้มีมั้ยครับ หรือคนที่เรียกร้องละมีมั้ย

2.ถ้าเป็นคนรู้กฎหมายก็อยากเรียกร้องอยู่ค่ะ แต่คนใรหมู่บ้านป้าก็แก่แล้วไม่ค่อยอยากเรียกร้องอะไร ถ้าเขาไม่ให้ก็โดนปรับอยู่ดี เหมือนเขาเรียกร้องเหมือนตากันในเฟสค่ะ เห็นแล้วไม่อยากเข้าไปกับเขา

1.รู้สึกไปก็สู้เขาไม่ได้หรอครับ

2.ใช่ค่ะ

1.อันนี้ก็เป็นผลกระทบนะครับ ขอเรื่องโควิดอีกนิดนะครับ ถ้าเราอยู่กับโควิดมาเป็นปีนี้นะ ป้าคิดว่าโควิดนี้เป็นโณคอะไร มันเกิดจากอะไร ในความคิดของป้าหน่ะครับ ไม่มีถูกไม่มีผิดนะครับ

2.ในความคิดของป้า มันคือยุคมัน ถ้าเป็นภาษาไทยใหญ่เป็นสมัยมัน

1.เหมือนกับว่าตอนเนี้ยมันจะมีโรคนี้เข้ามา แบบนี้หรอครับ

2.มันเหมือนคนเรานี้แหละ เหมือนสมัยของป้า ตอนนั้นมันก็ไม่ค่อยมีอะไร โรคก็ไม่ค่อยมี เสื้อผ้าก็ไม่ค่อยมีใส่ โทรศัพท์ก็ม่ค่อยรู้จัก อันนี้ก็มีมา โทรอย่างเดียว พอเดี๋ยวนี้มีการเห็นหน้ากันด้วยค่ะ โรคก็เป็นแบบนี้ค่ะ มันก็มาตามสมัยมันค่ะ

1.ป้ารู้สึกว่าไม่สบายใจ กลัวติดเชื้อบ้างมั้ยครับ

2.กลัวค่ะ กลัวเพราะว่ามันไม่เห็นตัวมันค่ะ ไม่รู้ใครติดใครค่ะ

1.โรคแบบนี้มันไม่ค่อนแสดงอาการออกมาให้เราเห็นด้วยตาเปล่าใช่มั้ยครับ

2.ใช่ค่ะ

1.แล้วที่กลัวในที่นี้คิดว่าเราเป็นกลุ่มเสียงด้วยมั้ยครับ หรือเสี่ยงไม่เสี่ยงไม่รู้ แต่ว่าโรคมันไปได้ทั่ว เรามองไม่เห็น ก็เลยคิดว่าเรากลัวจะติดเชื้อมั้ยครับ เพราะป้าเป็น อสม ไปหาคนนั้นคนนี้ โอกาสเสี่ยงก็มีมั้ยครับ

2.ก็เสี่ยงอยู่นะ ใครมาจากไหนไม่รู้ ตอนนี้ก็มีกฎขึ้นมาว่า ถ้ามีคนมาแล้วไม่กักตัวก็จะมีการปรับ

1.อันนี้เป็นกฏในชุมชนเลยหรอครับ

2.กฏในอำเภอเราหรือป่าว ตอนนี้ลดลงหน่อย ถ้าใครมาก็ต้องรายงานตัวที่อนามัยที่ผู้ใหญ่บ้าน ถ้าใครมาจากพื้นที่เสี่ยงก็ต้องกักตัวไว้ ตอนนี้ก็สบายใจขึ้นหน่อย

1.ต้องใช้กฏหมายมาขู่ถึงจะทำใช่มั้ยครับ ถ้าขอความร่วมมือนี้มีใครทำมั้ยครับ

2.ไม่ค่อยจะทำค่ะ

1.จากที่เป็น อสม มายี่สิบปีตตรวจลูกน้ำยุงลายขอความร่วมมือไม่ค่อยจะทำ ใช่มั้ยครับ

2.ต้องมีปรับค่ะ ปีที่แล้วหน้าฝนเป็นเยอะมาก เอายังไงก็ไม่อยู่ เอาไปเอามาให้ไปทุกหลังคาเรือนก่อนไปบ้านคนอื่นให้ดูแลบ้านตัวเองก่อน ปีนี้ไม่ค่อยมีแล้ว

1.ช่วงโควิดนี้ต้องดูแลยังไงครับ

2.โควิดนี้ไม่ค่อยเท่าไหร่ค่ะ โรคมันน่ากลัวมาก ต้องให้หัวหน้าอนามัย รอบแรกป้าได้ไปอยู่ไปวัดไข้ รอบนี้ไม่ได้ไป ต้องรายงานผู้ใหญ่บ้านไว้ ถ้ามาถึงให้มารายงานตัวกับอนามัย ถ้ามาจากที่เสียงสูง ก็ให้ไปตรวจดู อสม ไม่ได้ไป ในรอบสอง รอบแรกช่วยกัน ป้าโดนด่าด้วย เขาลือกันในหมู่บ้าน ว่าคนในหมู่บ้านป้ามา 2 คน แต่เขาแจ้งคนเดียว ป้าถึงมารู้เลยต้องแจ้งในกลุ่มไลน์ ป้าก็ไม่ได้โกรธอะไร เขาด่าเราเหมือนเราไปยุ่งเรื่องเขา

1.คนรู้จักกันนี้หรอครับ

2.ใช่ค่ะ

1.บั่นทอนจิตใจไหมครับคนทำงาน

2.ไม่ค่ะ ไม่กลัว เราทำตามหน้าที่ค่ะ อสม บางก็ไม่เข้มงวดค่ะ

1.ป้าคิดว่าป้ามีโอกาสเสี่ยงที่จะเป็นโรคมั้ยครับ สำหรับตัวป้าเอง

2.คิดอยู่ค่ะ จะมาถึงเรามั้ย คนบ้านเราไปทำงานต่างจังหวัดก็เยอะ บางคนไปทำงานก็ไม่กักตัว ก็กลัวอยุ่นะ เข้าตลาดก็กลัว เพราะว่าโรคก็ไปทั่ว คนบ้านเราก็ไม่ค่อยรู้เรื่อง

1.กับอีกอย่างหนึ่ง ป้ากำลังองว่าสำหรัยคนพื้นที่สูงสำหรับมาตรการต่างๆที่เขาห้ามด้วยภาษาด้วยอะไรเขาไม่รู้เรื่อง เขาก็ใช้ชีวิตปกติ เขาให้กักตัวก็ไม่กักตัว เพิ่มโอกาสเสี่ยงเข้าไปอีก

2.แต่สำหรับรอบที่สองก็มีกักตัวอยู่กักตัวที่บ้าน คนที่ไม่ค่อนเสี่ยงเท่าไหร่ก็ให้กักตัวที่บ้าน แต่ก็อยู่ด้วยกันอยู่ดีค่ะ

1.แสดงว่ามาตรการกักตัวนี้ยังไม่ค่อยประสบความสำเร็จใช่มั้ยครับในความรู้สึกป้า แม้กระทั่งรอบสองนี้

2.ใช่ค่ะ แต่รอบสองไม่ค่อยเท่าไหร่ ผู้ใหญ่บ้าน อำเภอเข้มงวดรู้ระบบมากขึ้น

1.แล้วป้าคิดว่าใครเป็นกลุ่มเสี่ยงที่จะติดโควิด

2.ทุกคนเลย

1.เมื่อปีที่แล้วละครับ คิดว่าใครเสี่ยง

2.ทุกคนค่ะ เพราะว่าไม่เห็นตัวมัน

1.โรคโควิดนี้ครับถ้าเทียบเท่าความรุนแรง ร้ายแรงมั้ยครับ ถ้าเทียบกับโรคที่เรารู้จักกันนี้ป้าเทียบกับโรคอะไรครับ

2.ร้ายค่ะ ยิ่งกว่าทุกโรคเลยค่ะ

1.ทำไมถึงคิดแบบนั้นละครับ

2.ก็เราไม่เห็นตัวมันค่ะ คนเราไปๆมาๆ ถ้าเขาไม่กักตัวยิ่งกว่านี้ แพร่เชื้อได้ง่าย

1.ที่เห็นว่ามันอันตราย การเห็นจากข่าวถือว่าเกี่ยวมั้ย มีส่วนไหมครับ เป็นแล้วตาย

2.ค่ะ เป็นแล้วบางคนก็รักษาได้ บางคนก็ไม่แน่ค่ะ ตอนนี้ก็มีข่าวว่าคนที่มีโรคประจำตัวก็เสียงสูง แต่ก็กลัวเหมือนกัน

1.ก็ถือว่ามันรุนแรงถ้าให้เรื่องระดับความรุนแรง

2.อันดับ 1 เลยค่ะ เบาหวานความดันเราคุมเองได้ค่ะ

1.แล้วป้าเชื่อมั้ยว่าการล้างมือ สวมหน้ากากอนามัย เว้นระยะห่างจะช่วยป้องกันโควิดได้

2.เชื่อค่ะ มันก็ป้องกันได้หลายอย่าง

1.ระหว่างกินร้อนช้อนกลางล้างมือใส่หน้ากากอนามัยอยู่ห่างกัน ป้าว่าอันไหนสำคัญสุด

2.ใส่หน้ากาก กับอยู่ห่างกันค่ะ

1.ป้าว่ามันเป็นอุปสรรคมั้ยครับ

2.ที่ยากนี้คือใส่หน้ากากค่ะ เพราะชอบลืม เลยทำยาก

1.คือให้ทำจริงๆนี้ไม่ยากใช่มั้ยครับ แต่จะยืม เวลาอยู่ด้วยกันก็คุยกัน ก็ลืมตัวใช่มั้ยครับ แล้วก็ป้าคิดว่าป้าสามารถได้ตลอดมั้ยครับ

2.ค่ะ ทำได้ค่ะ รอบที่แล้ว ถ้าจะเข้าตลาดไม่ใส่ไม่ได้ คนคัดกรอง รอบนี้ไม่มี

1.แล้วก่อนการระบาดเราดูแลสุกภาพยังไง

2.ดูแลปกติค่ะ

1.แล้วพฤติกรรมกินร้อนช้อนกลางนี้มีอยู่แล้วมั้ยครับ

2.มีอยู่แล้ว ทำอยู่

1.ระหว่างการระบาดระครับ หน้ากาเพิ่มขึ้น รักษาระยะห่าง ล้างมือ เจลล้างมือต้องซ้อกันมาเลยหรอครับ

2.รอบที่ 1 เราไม่เคยได้ยิน มันน่ากลัวมาก เราก็มีทุกบ้านค่ะ พกใส่กระเป๋าค่ะ

1.แล้วเรามีส่วนร่วมในการควบคุมโรคนี้อย่างไรบ้าง

2.เป็น อสม ค่ะ

1.แล้วปฏิบัติตามข้อแนะนำใช่มั้ยครับ

2.ค่ะ เขากลัวโรคติดเรา

1.ถ้าป้าไม่สบายมีอาการคล้ายโควิด ป้าจะทำอย่างไรครับ สมมตินะครับ

2.ต้องรีบไปหาหมออย่างเดียวค่ะ อาทิตย์ที่แล้วลูกเป็น เป็นรุนแรงมาก ก็รีบไปหาหมอ หมอบอกยังไม่เป็นหรอกป้า เพราะบ้านเรายังไม่มีคนเป็นเลย

1.หลักๆคือป้าใช้วิธีการไปหาหมอก่อนเลยนะครับ

2.ค่ะ เพราะบ้านเราก็ไม่ไกลอนามัยค่ะ

1.ป้าเคยได้ยินข่าวมั้ยครับว่าจะมีวัคซีน

2.ค่ะ ได้ยิน

1.บางคนต่างประเทศก็เริ่มฉีดกันแล้ว บางคนตายบ้างก็มี

2.ค่ะ ฉีดแล้วตายไม่เคยได้ยินค่ะ

1.แต่ก็เคยได้ยินว่าจะมีวัคซีนใช่มั้ยครับ

2.ค่ะ

1.ป้าคิดว่าวัคซีนจะช่วยตัวป้าเองได้มั้ยครับ จะช่วยให้ห่างจากการติดโรคโควิดมั้ยครับ

2.ก็ไม่เคยเห็นว่ามมันจะช่วยได้หรือป่าว ไม่แน่ใจ เพราะไม่เคยมีในไทย

1.ถ้าสมมติเดือนหน้าไทยมีวัตซีนนำเข้าจากอเมริกาแล้วเอาให้คุณป้า ให้ป้าให้ประชาชน ป้าคือหนึ่งในนั้น ป้าจะฉีดมั้ยครับ

2.ไม่กล้าฉีดค่ะ กลัวเป็นหนูทดลอง ถ้าสมมิตมาป้าก่อน ป้าจะไม่กล้าฉีดก่อน กลัวติด

1.ถ้าวัคซีนตัวนั้นเขาพัฒนาวัคซีนเรียบร้อยแล้ว เชิญป้ามาฉีดยา

2.จะฉีดอยู่ ขอรอดูผล ถ้าผลดี ผ่านการทดลองหลายขั้นตอนจะฉีดอยู่

1.ป้าคิดว่าตัวป้าเองมีโอกาสที่จะได้ฉีดวัคซีนมั้ย

2.ไม่แน่ใจค่ะเพราะอาจจะมีโอกาสก็ได้นะ

1.ถ้าวัคซีนออกมาเขาจะให้ใครฉีดก่อน

2.ป้าไม่รู้ ป้าบอกไม่ได้ ไม่รู้เรื่องว่าเขาจะจัดสรรให้ใคร

1.รู้แต่ว่าถ้าจะมาหาป้า แล้วเชิญป้าเป็นหนูทดลองไม่เอา แต่ถ้ามันดีแล้วมั่นใจแล้ว อันนั้นจะเอา ป้ารู้สึกมีความกังวลหรือกลัวการฉีดวัคซีนโรคโควิดมั้ยครับ

2.ไม่กังวล

1.แต่ก็มีเงื่อนไข ถ้าจะฉีดก็ต้องให้มั่นใจ มั้ยครับ แต่ถ้าดูแล้วยังไม่มั่นใจก็ขอดูก่อน ถ้ามีวัคซีนเรียบร้อยแล้วว่ามาฉีดวัคซีนกันในที่ป้าที่เป็น อสม และประชาชนคนหนึ่ง ป้าอยากมีส่วนร่วม รณรงค์มั้ย

2.ถ้ามันโอเคแล้วทำ

1.ถ้าวัคซีนโอเคแล้วทำ อย่างน้อยก็เชิญชวนในเขตพื้นที่ที่เรารับผิดชอบ ใช้วิธีการบอกต่อเนาะ แล้วความคาดหวังละครับ

2.อยากให้โรคนี้จบเร็ว ให้มันหายไปเลย ความคิดของป้านะ คิดว่ามันจะหายไปเองหรือป่าวหรือว่าจะเป็นไปเรื่อยๆ แค่วัคซีนน่าจะผลิตได้สำเร็จเพราะกระทบทั่วโลก

1.คาดหวังอะไรจาดภาครัฐ อยากให้เขาช่วยอะไร

2.อยากให้ช่วยเร่งเศรษฐกิจ ก็ให้ปรับปรุงบ้านเมืองเหมือนเดิม ตอนนี้ สมมติป้าไปแม่สาย แม่สายไม่มีคนเลย เหมือญาติจะมาหาก็มาไม่ได้

1.ค้าขายแย่ไปหมดเลยนะครับ แล้วบุคลลากรทางการแพทย์ ป้าต้องการความช่วยเหลืออะไรบ้างจากเขาครับ

2.หมอ พยาบาลทำงานหนัก เขาก็เป็นกลุ่มเสี่ยงกว่าเราค่ะ

1.เพราะฉะนั้นอยากเรียกร้องอะไรจจากเขาไหม

2.ไม่อยากค่ะ สงสารเขา อยากให้กำลังใจเขา ถ้าเขาติด เขาก็ติดก่อนเรา ตรงนั้นมีแต่คนเป็นโรค

1.ป้ามองว่าการที่เราอยู่บ้านนี้ก็ดีกว่าเขาแล้วใช่มั้ยครับ

2.ใช่ค่ะ เหมือนคนที่ทำงานอยู่ทุกวัน คนกักตัวก็ต้องไปดูแลทุกวัน ถ้ากลุ่มเสีย่งมาก็ต้องไปดู เสี่ยงกว่าเราเยอะ

1.ความคาดหวังต่อชุมชนบ้านเราละครับอยากให้คนในบ้านเราเป็ยอย่างไรในโรคโควิด อยาให้เขาปฏิบัติตัวอย่างไร ป้องกันอย่างไร

2.อยากให้เขาป้องกันตัวแบบใส่หน้ากาก อยู่ห่างงกัน บางคนไม่รู้เลย มีงานรอบสองก็เพิ่งมา เลยไม่มีคนใส่แมส

1.ที่เขาไม่ใส่นี้เขาหายใจไม่ออกหรือป่าว เขาเคยมาบอกป้าไหมครับ

2.เขาไม่รู้ แล้วก็ลืมบ้าง ส่วนมากจะลืม เพราะเราไม่เคยใส่กัน

1.เขาให้กักตัวก็ให้กักตัวสะ สิ่งที่ป้าคาดหวัง โควิดอยู่กับเรามาเป็นปีแล้ว เรารู้อะไรในการอยู่ร่วมกับโควิดบ้างส่วนตัวป้าเอง

2.ก็ทำให้เราปฏิบัติตัวป้องกันได้หลายโรค

1.เช่นโรคอะไรบ้างครับ

2.ไวรัสตับอักเสบ แล้วก็สมมติทำให้เราไม่รังเกียจกัน อย่างเราใช้ช้อนกลางนี้ใครมากินด้วยก็ได้ แต่ถ้าเราใช้ช้อนตัวเองคนก็รังเกียจกัน

1.อันนี้ธรรมชาติทั่วไปเลยเนาะ จะเป็นโรคไม่เป็นโรคใครก็รังเกียจ เราก็ต้องใช้ช้อนใครช้อนเรา อันนี้เกิดจากประสบการณเลยหรือคุณหมอสอนมาที่เราป้องกันโรคนี้ได้

2.ป้าเป็น อสม แล้วก็ได้ยินหมอเขาบอก ทำตาม แล้วก็เห็นคนกิน บางคนเป็นโรคแต่ไม่แสดงอาการ

1.ป้าคิดว่าปีนี้โควิดจะหายไปหมดไหม

2.ใกล้สิ้นปีโน้นแหละเนาะ อาจจะวนไปวนมาอยู่แบบนี้ ยิ่งกว่าผีอีกเราก็ไม่เห็นตัวมัน แต่มันก็ไม่ได้มาทำอะไรเรา แต่อันนี้มันไม่ได้ เราก็ต้องทำมาหากินทุกวัน ร้ายกว่าผี

1.ป้าคิดว่าผีมันไม่ทำ

2.ถ้าเราไม่ไปทำอะไรมัน มันก็ไม่มาทำอะไรเรา

1.แต่โควิดนี้ละ

2.อย่างถ้าเราไปเซเว่น เราก็ไม่รู้ว่าใครไป ใครมา

1.สุธิมา หลากอารีย์ ขอเบอร์โทรคุณป้าไว้ได้ไหมครับ

2. 097 9957824

1.แต่งงานแล้วนะครับ มีลูกกี่คนครับ

2.คนเดียวค่ะ

1.วันนี้ก็สัมภาษณ์เสร็จแล้วนะครับ ขอบคุณครับ

1. ผู้สัมภาษณ์ 2. ผู้ถูกสัมภาษณ์

**P2 ยายแก้ว.m4a**

1.คุณยายชื่ออะไรครับ

2.ยายแก้ว คำอู

1.อายุเท่าไหร่ครับ

2. 70 ค่ะ

1.คุณยายโรคโควิดนี้ส่งผลกระทบต่อคุณยายไหมครับ

2.ไม่

1.ไม่ได้รับผลกระทบหรอ ทำไมหรอครับ

2.ไม่รู้ ปกติยายก็ไม่ได้ไปไหน คงมากับคนอื่นค่ะ

1.ยายคิดว่าไม่ได้รับผลกระทบ ยายก็ใช้ชีวิตได้ตามปกติ ไปพบลูกหลาน

2.ลูกหลานไม่มา ลูกหลานอยู่กรุงเทพ

1.ลูกหลานจะมาหาเอง หรือว่าเขาไม่ให้มา

2.เขาไม่ให้มา

1.แล้วไม่รู้สึกคิดถึงหรอ

2.ก็คิดอยู่ เขาก็โทรมา

1.แล้วเรื่องรายได้ละครับ ได้รับผลกระทบไหม

2.ก็อยู่บ้านทำกับข้าวลูกหลานไปทำงานก็ส่งตังมาให้ใช้ ปลูกผักกิน

1.มีลูกกี่คนครับ

2. 5 คน

1.อยู่ที่บ้านเรามีกี่คน

2.มีหลานคนหนึ่งกับลูกสะใภ้ 1 คน

1.อ่ออยู่กับหลานหรอครับตอนนี้ หลานเลี้ยงหรอครับ

2.หลานอยู่บ้าน ลูกก็ไปทำงานกรุงเทพ

1.หลานเป็นลูกของลูกคนที่เท่าไหร่ครับ

2.คนที่ 2

1.เงินรายได้ของยายได้มาจากไหน ได้เบี้ยผู้สูงอยุไหมครับ

2.ได้

1.ได้จากไหนอีกไหมครับ ลูกส่งให้ หรือมีรายได้ของตัวเองครับ

2.ลูกส่งให้ ซื้อกิน ทำบุญ

1.เบี้ยผู้สูงอยุตอนนี้ได้เท่าไหร่แล้วครับ

2.จะได้ 700 แล้ว

1.ลูกก็ส่งให้ด้วยนะครับ แล้วอาชีพหลักๆ ทำนาเย็บผ้า ยังทำอยู่ไหมครับ

2.ไม่ได้ทำ ให้ลูกหลานทำให้

1.วิถีชีวิตเปลี่ยนไปไหมตั้งแต่มีโควิด ที่เขาบอกว่าให้ใส่หน้ากาก ให้อยู่ห่างกัน ให้ล้างมือบ่อยๆ รู้สึกไหมว่าเราปฏิบัติตัวยากไหมครับ

2.ก็ปฏิบัติปกติแหละ ทำได้

1.ยายทำได้ใช่ไหมครับ รู้สึกว่าหายใจไม่ออกบ้างไหนครับ

2.ก็ใส่ตอนเจอกัน อยู่คนเดียวก็เอาออก

1.เคยลืมหน้ากากไหม

2.ก็ใส่กระเป๋าไว้ ตอนแรกๆก็ลืมอยู่

1.ลูกหลานไม่ได้กลับมานานหรือยังครับ

2.นานแล้ว

1.ปกติลูกหลานจะกลับช่วงไหนครับ สิ้นปีหรือสงกรานต์

2.สงกรานต์

1.สงกรานต์ปีที่แล้วก็ไม่ได้กลับ เขาไม่ให้กลับเนาะ แล้วปีใหม่เมื่อกี้ละครับ

2.ปีใหม่ก็ไม่ได้กลับ ถ้ามาต้องกักตัว

1.ใช้วิธีการโทรศัพท์ เอา โทรหาเอานะครับ เรื่องการเข้าถึงบริการสุขภาพ คิดว่าลำบากไหมครับในชาวงที่มีโควิดระบาดใหม่ ยายเป็นเบาหวานความดันไหมครับ

2.ไม่ เบาหวานก็ไม่มี ความดันมีนัดกับหมอในสองสามวันนี้ แต่ก่อนเป็น หมอให้ยามากิน

1.พอมีโควิดหมอห้ามไม่ให้มาโรงพยาบาลมีบ้างไหนครับ

2.ไม่ห้าม เขาบอกยาหมดให้ไปเอายา

1.ยายเคยได้ยินโรคโควิดไหมครับที่เราเรียกอยู่ทุกวัน เกิดจากอะไร

2.ไม่รู้

1.ยายว่ามันเป็นโรคติดต่อไหมครับ ยายคิดว่ายายจะติดโรควิดไหมครับ

2.ไม่อยากติด

1.ยายว่าโรคโควิดรุนแรงไหมครับ เป็นแล้วตายไหมครับ

2.เป็นแล้วตายเลย

1.ยายรู้ได้ไงว่าเป็นแล้วตาย

2.เขาว่ามาว่าถ้าเป็นแล้วก็ตายเลย ฟังมา

1.ยายเชื่อว่าโรควิดมันน่ากลัวไหม น่ากลัวยังไง

2.น่ากลัว ก็คนที่ติดนี้บุญใครบุญมัน กรรมใครกรรมมัน

1.คนที่ไม่ป่วยนี้ก็ทำบุญมาดีหน่อยใช่ไหมครับ แล้วยายเคยได้ยิน กินร้อน ช้อนกลาง ล้างมือ บ่อยๆ ให้ใส่หน้ากาก ให้อยู่ห่างกัน ยายเคยได้ยินไหมครับ

2.ได้ยิน เขาประกาศ ยายก็ได้ยินประกาศเสียงตามสายในหมู่บ้าน

1.ที่เขาประกาศนี้ยายได้ทำตามไหมครับ

2.ไม่ได้ทำ

1.ที่เขาบอกว่าให้กินร้อน ช้อนกลาง ล้างมือ บ่อยๆ ให้ใส่หน้ากาก ให้อยู่ห่างกัน ยายไม่ได้ทำหรอครับ

2.ทำอยู่ๆ

1.ขอโทษนะครับยายนับถือศาสนาอะไรครับ

2.ศาสนาพุทธ

1.แล้วช่วงหนึ่งเขาห้ามไปวัด ยายทำไง

2.ยายก็ไปอยู่ ไปทำบุญไม่เป็นอะไร ไปไม่นาน รีบไปรีบกลับ ชอบทำบุญ ชอบใส่บาตร

1.แล้วทำยังไง เอาของไปให้ใคร

2.ขอศีลขอพร แล้วก็กรวดน้ำ ไหวพระพุทธองค์แล้วก็กลับมา

1.ยายคิดว่าถ้าเราปฏิบัติตามมาตรการกินร้อนช้อนกลางล้างมือใส่หน้ากากจะป้องกันโรคโควิดได้ไหมครับ

2.ได้ค่ะ

1. แล้วมันทำยากไหมครับ

2.ก็อดทนเอา

1.ตอนแรกๆก็งงๆหน่อยใช่ไหมครับ ตอนนี้ดีขึ้นมาหน่อย

2.เข้าใจแล้ว แต่ก่อนยังงง ใส่หน้ากาก ตอนนี้อดทนก็ได้ ใส่หน้ากากป้องกันโรค

1.ตอนนี้ปรับตัวได้แล้วติดสะว่าป้องกัน ก็ต้องอดทน คิดว่าทำแล้วลำบากไหมครับ

2.ทำได้ทุกอย่าง

1.เรื่องลูกหลานไม่ได้กลับมาตอนแรกก็คิดถึง ก็โทรเอา โควิดอยู่กับเราปีที่สองแล้วนะครับก็เริ่มชินแล้ว ก้โทรคุยกันเอาใชช่ไหมครับ แล้วยายเคยได้ยินไหมว่าเขาจะมีวัคซีน เคยได้ยินข่าว หรือได้ยินใครพูดไหมครับ

2.มีได้ยิน

1.แล้วผมจะถามยายว่าวัคซีนจะช่วยเราป้องกันโควิดได้ไหมครับ

2.ไม่รู้ แต่รู้ว่าถ้าเขามาฉีดก็ดี คิดว่าเป็นยาที่ดี

1.แล้วถ้าสมมติประเทศไทยคิดค้นวัคซีนป้องกันโรคโควิดแล้ว แล้วเขาเชิญคุณยายมาฉีด คุณยายจะฉ๊ดไหม

2.ถ้าคุณหมอเชิญไป ก็ไปอยู่

1.แล้วถ้าสมมติผมเป็นหมอ แล้วจะขอฉีดวัคซีนป้องกันโรคโรควิดให้ยาย ยายจะยอมไหมครับ

2.ให้ฉีดอยู่

1.เพราะอะไรถึงยินดี

2.ก็หมอก็ป้องกันเรานะ ป้องกันไว้ เชื่อหมอ เป็นยา ก็เชื่อหมอ

1.เชื่อหมอ หมอว่าดีก็ดี เวลาเราจะฉีดวัคซีนเราจะกังวลไหมว่าจะทำให้เราไม่สบาย

2.ไม่กลัว

1.ถ้าฉีดวัคซีนยายคาดหวังให้สุขภาพเราเป็นยังไง

2.ฉีดแล้วก็ป้องกันเรานะสำหรับผู้สูงอายุจะแข็งแรงดี หมอก็รักษา

1.ถ้าสมมติมีวัคซีนเรียบร้อยยายอยากมีส่วนร่วมในการบอกลูกหลานคนแก่แถวบ้านไหมครับว่าให้ไปฉีดวัคซีนนะ

2.จะช่วยบอกอยู่

1.ที่ผ่านมาก่อนหน้าที่จะมีโรคโควิดระบาดยายมีวิธีดูแลตัวเองอย่างไรบ้างครับ

2.ยายก็ดูแลตัวเอง เขาบอกมาอย่างนี้ ต้องอย่างนี้ๆ คนแก่ก็ต้องทำบุญถือศีล บุญใครบุญมัน ส่วนการรักษาตัวถือศีบทำบุญ

1.ก่อนหน้าจะมีโควิดยายได้ล้างมืออยู่เป็นประจำไหมครับ

2.ทำๆเป็นประจำ

1.กินข้าวนี้ช้อนใครช้อนมันไหมครับ กับข้าวที่อยู่ตรงกลางนี้มีช้อนกลางไหมครับ

2.มีช้อนกลาง

1.ทำมานานหรือยังครับ

2.ยายก็อยู่บ้าน หลานก็ไปโรงเรียน กินคนเดียวนั้นแหละ กินผักเนื้อสัตว์ไม่กิน

1.ทำมานานหรือยังครับ

2.ทำมาได้สี่ปี

1.ระหว่างที่เกิดการระบาดยายปฏิบัติตัวตามมาตรการดีไหมครับ

2.ก็ปฏิบัติดีอยู่

1.ช่วงนี้ถ้าเขามาขอความร่วมมือว่าอย่าออกไปไหนนะโควิดระบาด ยายก็ให้ความร่วมมืออยู่ใช่ไหมครับ

2.ก็ให้ความร่วมมือ

1.นานๆทียายไดไปตัวเมืองเชียงรายบ้างไหมครับ

2.เชียงรายยายก็ไปนานแล้ว ไม่ได้ไป อยู่แต่บ้านเป็นหลัก

1.แล้วคนในชุมชนมีบ้างไหนครับที่ลูกหลานเขาไปทำงานกรุงเทพแล้วกลับมา

2.ที่ยายอยู่นี้มี

1.แล้วที่ยายรู้มาว่าคนที่ไปทำงานกรุงเทพกลับมาแล้วเขากักตัวไหม

2.ไปหลังวัด

1.แล้วคนที่ไม่กักมีไหม

2.คนที่ไม่กักไม่มีค่ะ

1.ยายคาดหวังความช่วยเหลืออะไรจากภาครัฐ อยากให้รัฐช่วยอะไรบ้าง

2.ก็อยากให้ช่วยรัฐบาลอยู่ไหนก็อยู่นั้น ถ้ากลับมาก็ทำให้มันถูกต้อง

1.อยากให้หมอ พยาบาล หมออนามัย อยากให้เขาช่วยเหลืออะไรบ้างไหนครับ

2.หมอก็อยากให้หมอช่วยสอนการรักษาตัว แต่หมอก็สอนอยู่ กินยารักษาตัวนะยาย

1.คนในชุมชนละครับ ยายอยากให้คนในชุมชนทำอย่างไรในโควิดนี้อยากให้ปฏิบัติตัวอย่างไรครับ

2.ผู้ใหญ่บ้านก็ประกาศบอกแต่คนก็ไม่ฟัง เราฟังเราก็จำไว้

1.แสดงว่ามีที่ฟังและไม่ฟัง แล้วคนที่ไม่ฟังก็มีหรอ

2.ยายก็ไม่รู้ แต่ยายฟัง

1.แล้วผูใหญ่บ้านเขาประกาศบ่อยไหนครับ

2.ทุกวันตอนเย็นไปกรุงดทพกลับมาก็ให้รักษาตัวนะ

1.ยายอยู่หมู่อะไรนะครับ

2.หมู่ 8

1.คุณตาเสียไปแล้วหรอครับ

2.ค่ะ

1.ยายเป็น อสมหรืออะไรแบบนี้ไหมครับ

2.อสม ไม่เคยเป็น

1.ยายได้เรียนหนังสือไหมครับ

2.ไม่ได้เรียน แต่เขียนชื่อได้อยู่

1.โรคโควิดที่มารอบนี้มันอยู่กับเรามาปีกว่าแล้ว มันสอนอะไรเราบ้าง ยายได้เรียนรู้อะไรจากมัน

2.มันสอนให้เรารักษาตัว ป้องกันตัวเอง ทำดีๆ ทำศีลทำบุญ ทำดีๆไว้ โรคภัยไข้เจ็บจะได้ไม่มาหาเรา

1.ยายคิดว่าโรคโควิดนี้จะอยู่ไปกับเราอีกนานไหมครับ

2.ก็อยากให้มันหาย สงสารลูกหลาน อยากให้เศรษฐกิจดีทำงานปกติลูกหลานจะได้กลับตัวกลับใจเป็นคนดี มีการมีงานทำ ลูกหลานก็ลำบาก

1.ลูกหลานโทรมาเล่าให้ฟังไหมว่าทำงานเป็นยังไง

2.ไม่ค่อยมี

1.ไปทำงานอะไรอยู่กรุงเทพครับ

2.งานก่อสร้าง

1.ปกติลูกหลานจะกลับมาหาช่วงไหนครับ

2.สงกรานต์ ปีใหม่ แต่นี้ไม่ได้ปีกว่าแล้ว

1.ผู้สัมภาษณ์ 2.ผู้ถูกสัมภาษณ์

**P4ยายซางเหมย.m4a**

1.คุณยายชื่ออะไรครับ

2.ซางเหมย แซ่โจว

1.อายุเท่าไหร่ครับ

2. 74 ค่ะ

1. ที่บ้านอยู่กับใคร

2.ลูกสะใภ้

1.คุณตาเสียแล้วหรอครับ

2.เสียแล้วค่ะ

1.ยายว่าโรคคิดเป็นโรคอะไรเคยได้ยินไหม

2.ไม่รู้

1.มันติดต่อยังไงรู้ไหม

2.ไม่รู้ว่าเป็นยังไง

1.เคยได้ยินแต่ไม่รู้ว่าคืออะไรใช่ไหมครับ รู้สึกว่าตัวเองติดโควิดไหม

2.ไม่ติดๆ

1.คิดว่าตัวเองเสี่ยงจะติดไหม

2.ไม่มีโอกาสติด

1.ในความคิดของคุณยายใครคือคนที่จะเสี่ยงติดโควิด

2.ตรงนี้ไม่มีก็ไม่รู้ค่ะ

1.โรคโควิดคิดว่าน่ากลัวไหม รุนแรงไหม

2.น่ากลัว

1.คิดว่าโรคนี้รุนแรง เป็นแล้วตายไหม

2.ตาย

1.คิดว่าโควิดนี้ถ้าเทียบความรุนแรงสามารถเทียบได้กับโรคอะไรครับ

2.น่ากลัวที่สุดมากกว่ามะเร็ง พวกนั้นยังยาสักรักษา เป็นแล้วตายเลย

1.การป้องกันโควิดนี้ต้องทำอะไรบ้าง

2.ใส่ผ้าปิดปากแล้วก็ขยันล้างมือให้สะอาดค่ะ อยู่ห่างกัน ไม่อยู่ด้วยกัน

1.คิดว่าทำได้ยากไหม

2.ไม่ยาก

1.ลืมหน้ากากไหม ตอนแรกๆ

2.ไม่ลืมค่ะ

1.ซื้อหน้ากากเองหรือลูกหลานซื้อมาให้ครับ

2.ลูกหลานฝากมาให้

1.แสดงว่ามีหน้ากากที่บ้านเยอะหรอ

2.มีหลายกล่องค่ะ

1.แล้วโควิดที่ผ่านมาตัวยายเองได้รับผลกระทบไหม เช่นลูกหลานไม่ได้มาหาเพราะเขาสั่งว่าไม่ให้เดินทางไปไหน

2.คนที่กรุงเทพนี้ไม่กลับมาเลย

1.มีลูกหลานอยู่กรุงเทพใช่ไหมครับ ยายมีลูกกี่คน

2. 4 คน

1. อยู่ไหนบ้าง

2. อยู่กรุงเทพ 2 คน อยู่นี้ 2 คน

1.อยู่กรุงเทพนี้ไม่ได้มาเลยนานกี่ปีแล้วครับ

2.ไม่มา ได้ปีสองปีแล้ว ไม่ให้กลับมาเพราะโควิด

1.ปกติลูกหลานมาหาช่วงไหน

2.สงกรานต์ค่ะมาไหว้เจ้า ที่มีโควิดนี้ไม่ให้มาเลยค่ะ ให้กักตัว

1.แสดงว่าตั้งแต่ปีที่แล้วที่ไม่ได้มา แล้วปีนี้คิดว่าจะได้กลับมาไหม

2.ไม่ให้กลับมาค่ะ

1.แล้วไม่คิดถึงลูกหลานหรอ

2.คิดถึงก็มีโทรศัพท์

1.เห็นหน้าด้วยไหม

2.เห็นหน้าค่ะ

1.ทางด้านสุขภาพร่างกายละ ตั้งแต่มีโควิด

2.ออกกำลังกายทุกวันที่นี้ เต้นรำ

1.มีโรคประจำตัวไหม เป็นโรคอะไรบ้าง

2.ความดัน เบาหวาน กินยาอยู่

1.ช่วงระบาดไปหาหมอที่อนามัยหรือโรงพยาบาลครับ

2.ไปโรงพยาบาลแม่ฟ้าหลวงค่ะ นัด 3 เดือนครั้ง

1.ทางด้านสุขภาพจิตใจละมีความกังวลไหมในช่วงที่มีการระบาดเราจะติดไหน ลูกหลานเราจะเป็นยังไง

2.นอนไม่หลับ คิดถึงเขาแต่ว่านอนไม่หลับ

1.นอนไม่หลับเพราะว่าอะไร กังวลแทนหรอ

2.คิดอะไรก็ไม่รู้ คิดไปเรื่อย

1.คิดตั้งแต่เริ่มมีโควิดนี้หรือครับ หรือเดิมเป็นคนนอนไม่หลับมาอยู่แล้ว

2.เป็นคนนอนยากอยู่แล้ว

1.ด้านเศรษฐกิจละ โควิดนี้เขาไม่ให้ไปนู้นไปนี้ ลูกหลานเราได้รับผลกระทบไหมครับที่อยู่กรุงเทพ

2.ไม่ให้ไป

1.รายได้ของยายลดลงไหมตั้งแต่มีโควิด

2.ลูกเขาก็ยังส่งเงินให้ใช้อยู่

1.ใครส่งตังให้ยายใช้

2.มีหลาน 2 คน ลูกของลูกชาย

1.ได้เงินผู้สูงอายุไหมครับ

2. ได้ 700 แล้ว

1.เงินพอใช้ไหม

2.ไม่ค่อยพอใช้

1.ไม่ค่อยพอใจแล้วทำยังไง ได้ยืมไหม ต้องกู้ไหม

2.ยืมค่ะ เงินของรัฐบาลก็ยืมค่ะ 40000 บาท

1.ตั้งแต่มีโควิดนี้ยืมมากกว่าเดิมไหมครับ

2.ลูกก็ให้ใช้

1.ยายเคยได้ยินเรื่องวัคซีนต้านโควิดไหม

2.ไม่เคยได้ยินค่ะ ที่ 60 ปี ไปฉีดมานี้วัคซีนอะไรคะ

1.ไปฉีดมาหรอยังครับ

2.ไปฉีดมาแล้ว

1.อันนี้พวกไข้หวัดใหญ่ทั่วไป แล้วเคยได้ยินไหน ว่าโควิดจะมีวัคซีนรักษาแล้วนะ

2.ไม่เคยได้ยินมาก่อนเลย ไม่รู้มาก่อน

1.ยายเชื่อไหมว่าวัคซีนไข้หวัดจะช่วยป้องกันให้โรคนี้หายไปเลย จะมีไหม

2.วัคซีนไข้หวัดใหญ่ๆ

1.แล้วถ้ามีวัคซีนให้กลุ่มเสี่ยงที่อายุมากว่า 60 ไปฉีดวัคซีนต้านโควิด ยายจะไปไหมครับ

2.ไปฉีด

1.กลัวไหนครับ ว่าถ้าฉีดไปแล้วจะเป็นโน้นเป็นนี้

2.ถ้ามีโรคประจำตัวเขาก็จะคงไม่ฉีดให้ใช่ไหมต้องตรวจก่อนถึงจะฉีดใช่ไหม

1.ใช่ครับเราก็ต้องตรวจดูก่อนครับ ถ้าสมมติมีวัคซีนมาให้ ให้คุณยายมาฉ๊ดคุยายจะมาฉีดไหม

2.ต้องดูก่อน

1.ถ้าวัคซีนมีเยอะๆ ยายติดว่ายายจะเป็นกลุ่มที่จะได้ฉีดไหมรัฐบาลหรือหมอจะเลือกยายไปฉีดไหม ในความคิดของยาย

2.ถ้าคนอื่นได้ฉีดเขาก็ฉีด

1.ดูที่คนอื่นก่อนนะ อย่างงี้เนาะเราอยู่บนดอย เราอยู่ที่สูง กับคนไทยข้างล่าง ยายคิดว่าระหว่างข้างล่างกับข้างบนใครจะได้ฉีดก่อน

2.คนข้างล่างค่ะ ข้างล่างฉีดก็ให้ข้างบนฉีด

1.ทำไมให้คนข้างล่างฉีดก่อน

2.เขาฉีดไม่กลัว เราก็ไม่กลัว

1.กังวลไหม กลัวไหม ว่ามีต่างประเทศบางที่ก็ฉีดแล้วไม่สำเร็จ ได้ดูข่าวไหม ฉีดแล้วตาย

2.ยายไม่รู้ข่าว คุยแต่โทรศัพท์

1.ถ้าเกิดว่าหมอมารณรงค์ให้ฉีดวัคซีนยายจะไปบอกคนข้างบ้านไหมว่าให้ไปฉีดวัคซีน

2.พูดให้เขาฟังว่ามีฉีดวัคซีน

1.ที่ผ่านมาโควิดนี้ต้นกำเนิดมาจากประเทศอะไรจำได้ไหม

2.เมืองจีนค่ะ

1.ก่อนหน้าจะเกิดโควิดยายออกกำลังเป็นประจำไหน

2.ออกทุกวันเลยค่ะ

1.ใส่หน้ากากไหมก่อนมีโควิด

2.ไม่ใส่

1.พอเกิดการระบาดยายปรับตัวยากไหม ที่ต้องล้างมือใส่หน้ากาก

2.ไม่ยากค่ะ

1.ทำไมเชื่อหมอว่าต้องใส่หน้ากาก ล้างมือ

2.กลัวโควิดค่ะ

1.ที่หมู่บ้านเรามีคนเสี่ยงที่จะเป็นโควิดไหม คนกลับจากกรุงเทพ ต่างประเทศ

2.ไม่คิด ไม่กล้าพูดไม่รู้ ใครเป็นใคร

1.ผู้ใหญ่บ้านขอให้ทำอะไรก็จะให้ความร่วมมือไหม

2.ทำอยู่ค่ะ

1.สมมติถ้าผมมาจากกรุงเทพ เป็นกลุ่มเสี่ยงมาบ้านเรา ผมต้องทำอย่างไรบ้างครับ

2.ต้องกักตัว กักที่โรงพยาบาล

1.ในชุมชนเรามีที่กักตัวไหม

2.กักที่บ้านตัวเอง

1.ต้องมีคนไปตรวจไหม

2.ไม่มี

1.ถ้าเขาออกมาเราจะทำไง

2.ไม่ให้ออกมา เอาข้าวไปให้

1.คนบ้านเราส่วนใหญ่นับถือศาสนาอะไรครับ

2.พุทธเยอะสุด คริสต์ อิสลามส่วนน้อย

1.คนชนชาติพันธ์มีชนชาติไหนบ้างครับ

2.จีน ลีซอ มูเซอร์ อาข่า ยายเป็นมุเซอร์

1.แล้วมีคนชนชาติอะไรเยอะครับ

2.เยอะสุดมูเซอร์ อาข่า สองอย่างค่ะ

1.ยายเชื่อว่าวัคซีนจะผลิตได้เร็ววันไหมในความคิดของยาย จะมีวัคซีนเกิดขึ้นไหม

2.อยากให้มาเร็วๆ

1.ผู้สัมภาษณ์ 2.ผู้ถูกสัมภาษณ์

**P3อำพร.m4a**

1.น้องชื่ออะไรนะครับ

2.อำพรค่ะ

1.อายุเท่าไหร่ครับ

2.อายุ 15 ปีค่ะ

1.ม.3 เรียนโรงเรียนอะไรครับ

2.เรียนโรงเรียนบ้านห้วยไทยค่ะ

1.น้องรู้จักโรคโควิดไหม แล้วเป็นยังไงในความเข้าใจของน้อง

2.โควิดคือเป็นเชื้อไวรัสที่น่ากลัว

1.น่ากลัวยังไง

2.ติดคนสู่คนทำให้เสียชีวิตค่ะ

1.ที่ผ่านมารู้สึกกังวลหรือไม่สบายใจไหมครับว่าเราติดหรือยัง

2.รู้สึกค่ะ เคยมีบ้าง

1.อะไรที่ทำให้รู้สึกแบบนั้นครับ

2.เหมือนเราเป็นหวัดเจ็บคอ

1.ก็คือมีอาการบางอย่าง เช่น

2.เจ็บคอ เป็นหวัด

1.แล้วเราทำยังไงต่อ คิดแล้วไปเสิร์ชต่อหรือคิดแล้วหายไปหรือไปหาหมอ

2.คิดแล้วก็เสิร์ชหาข้อมูลแล้วก็บางทีก็กินยาบางทีก็ไม่กิน ยาลดไข้ค่ะ

1.แล้วมันก็หายไป แต่ไม่ถึงขั้นไปหาหมอ

2.ไม่ถึงค่ะ

1.แต่มีคิดอยู่ว่าติดหรือยังใช่ไหมครับ แล้วคิดว่าเรามีโอกาสเสี่ยงที่จะติดโรคโควิดบ้างไหมครับ

2.ติดได้ค่ะ เพราะถ้าเราไปในพื้นที่เสี่ยงก็จะติดได้

1.แล้วหนูคิดว่าหนูไปไหม

2.ไม่น่าจะไปค่ะ

1.แล้วหนูอยู่แถวบ้านทั้งบ้านโรงเรียนมีโอกาสได้เข้าไปในเมืองไหมครับ

2.ถ้าไปก็ใส่หน้ากาก แมส แล้วก็ล้างมือบ่อยๆค่ะ

1.หนูคิดว่าใครคือกลุ่มเสี่ยงของโรคนี้

2.คนที่มาจากต่างประเทศ เดินทางบ่อยๆในพื้นที่เสี่ยง

1.พื้นที่เสี่ยงที่หนูเข้าใจนี้มีที่ไหนบ้างครับ

2.ที่คนเยอะๆ

1.ถ้าเปรียบเทียบความรุนแรงหนูคิดว่าโรคโควิดมีความรุนแรงมากไหมครับ

2.มากค่ะ ถึงขั้นตายได้เลย

1.ถ้าเปรียบเทียบโรคโควิด หนูจะเปรียบเทียบเท่ากับโรคอะไร ในความรุนแรงตามที่หนูเข้าใจ

2.โรคมะเร็งค่ะ

1.ความรุนแรงของโควิดนอกจากเป็นแล้วตายยังมีความรุนแรงอย่างไรอีก

2.ถ้าไม่ตายก็น่าจะเสียสุขภาพ

1.เสียสุขภาพนี้คืออวัยวะหรือยังไงครับ

2.พวกปอดไม่ทำงาน ไม่ดี ร่างกายน่าจะไม่ร้อยเปอร์เซ็นค่ะ

1.เกี่ยวกับพฤติกรรมสุขภาพละครับ เชื่อไหมเรื่องการกินร้อนช้อนกลางล้างมือใส่แมสเว้นระยะห่างกันไม่ไปพื้นที่เสี่ยงสามารถป้องกันโควิดได้ เชื่อไหม

2.ป้องกันได้ค่ะ เพราะว่าถ้าใช้ของร่วมกันก็สามารถที่จะติดกันได้ ถ้าล้างมือป้องกันอย่างดีก็จะไม่เป็นค่ะ

1.แล้วมาตรการกินร้อนช้อนกลางล้างมือใส่แมสเว้นระยะห่างกันไม่ไปพื้นที่เสี่ยงอันไหนดีที่สุดเลยในตอนนี้

2.ก็คือการล้างมือแล้วก็ใส่แมสค่ะและไม่ไปที่คนเยอะๆ

1.ทำไมถึงเชื่อว่าพวกนี้ป้องกันได้มากกว่ากินร้อนช้อนกลาง

2.ไม่รู้ค่ะ

1.อุปสรรคละ เราก็รู้ว่าวัคซีนยังไม่ประสบความสำเร็จ เขาก็มีการรณรงค์ไม่ให้ไปพื้นที่เสี่ยง ให้กักตัว ให้ใส่แมส สิ่งพวกนี้ทำยากไหม เป็นอุปสรรคไหมครับ

2.ไม่ยากค่ะ

1.ยังไงครับ

2.ก็ต้องคิดว่าถ้าไม่อยากติดก็ต้องทำค่ะ มันเป็นสิ่งที่ต้องป้องกันค่ะ

1.ไม่ยากในที่นี้คือแมสหาง่ายไหม ซื้อง่ายไหม

2.ได้ค่ะ

1.การเว้นระยะห่างเขามีประกาศไม่ให้ไปโรงเรียนบ้างไหนครับ

2.ไม่มีค่ะ อาจจะบ้างที่เขาประชุมแต่ไม่มี

1.แล้วเรียนออนไลน์มีบ้างไหนครับ

2.มีค่ะ

1.ต้องทำอย่างไร

2.ต้องซื้อเน็ตเพิ่มค่ะแล้วก็ไม่เหมือนไปเรียนที่โรงเรียน เรียนไม่เข้าใจเท่าไปโรงเรียนค่ะ

1.เกรดดีไหมที่ผ่านมา

2.ไม่น่าดีค่ะ

1.ส่วนตัวหนูหนูเชื่อว่าสามารถทำตามมาตรการได้หมดไหม

2.ทำได้ค่ะ

1.ถ้าเต็ม 10 หนูให้คะแนนตัวเองเท่าไหร่ครับ

2.8 ค่ะ

1.ด้านไหนที่หนูคิดว่าทำได้ที่สุด

2.คงจะเป็นการล้างมือแล้วก็ไม่ใช้ของร่วมกับคนอื่นๆใส่แมส ไม่ไปไหนมาไหน ไม่ไปในพื้นที่เสี่ยงค่ะ

1.แล้วด้านไหนที่ดูคิดว่าให้คะแนนแล้วยังไม่ค่อยผ่านที่สุด

2.อาจจะหลีกเลี่ยงคนได้ไม่เยอะค่ะ

1.ทำไมถึงหลีกเลี่ยงไม่ได้

2.เพราะว่าแต่ละคนเขามองไม่เหมือนกัน

1.ผลกระทบทางด้านสุขภาพ ตั้งแต่มีโควิดเข้ามาทำให้หนูได้รับผลกระทบในด้านนั้นไหมครับ ทั้งด้านร่างกายและจิตใจทั้งความคิดวิตกกังวลมีบ้างไหนครับ

2.ก็กลัวอยู่ค่ะ เพราะโรงพยาบาลเป็นพื้นที่เสี่ยง

1.รู้สึกวิตกกังวลนะครับ ทางด้านสังคมละครับ เดินทีที่เราไปหาเพื่อนกอดคอกันได้ตอนนี้เขาบอกอย่าเพิ่ง รู้สึกว่ามันขัดต่อวิถีชีวิตเราไหมครับ

2.ได้รับค่ะ ไม่ได้ไปเที่ยวแล้วก็ไม่ได้ออกไปไหนค่ะ ไปซื้อของก็ลำบากค่ะ

1.ปกติเวลาไปเที่ยว ไปเที่ยวไหนครับ

2.เข้าในเมืองค่ะ

1.ขับมอไซต์หรือเอารถใหญ่ไป

2.เอามอไซต์ไปกับเพื่อนบ้างแล้วก็ไปกับครอบครัวบ้างค่ะ

1.มีเพื่อนหรือญาติอยู่ต่างจังหวัดไหม แล้วเขาไม่ได้กลับมา

2.ใช่ค่ะ ไม่ได้กลับมาเลยค่ะ

1.ใครไปทำงานต่างจังหวัดครับ

2.พ่อแม่ค่ะ

1.แล้วตอนนี้หนูอยู่ที่บ้านกับใครครับ

2.อยู่กับตายายค่ะ

1.พ่อแม่ไปทำงานที่ไหนครับ

2.กรุงเทพค่ะ

1.ไม่เจอกันเป็นปีแล้วมั้ง

2.ไม่เจอเลยค่ะ

1.แล้วใช้วิธียังไงครับ

2.โทรศัพท์คุยกันเอาค่ะ

1.ด้านเศรษฐกิจสังคมละครับ ตอนนี้หนูอาจจะยังเป็นนักเรียนยังไม่คิดวิตกกังวลมาก แต่ด้านการเงินอาจจะกระทบต่อผู้ปกครองมีกระทบมาถึงเราบ้างไหมครับ

2.ส่งมาน้อยบ้างแล้วก็ขาดแคลนบ้าง

1.ขอโทษนะครับ พ่อแม่นี้ไปทำงานอะไรครับ

2.รับจ้างทั่วไปค่ะ

1.พ่อแม่เคยบ่นๆไหมครับเรื่องที่จากเดิมเคยมีงานเยอะแล้วตอนนี้ก็มีน้อยลง

2.มีค่ะ

1.วิถึชีวิตของหนูละ ดิดว่าตั้งแต่มีโควิดมาการใช้ชีวิตหนูเปลี่ยนไปไหน

2.เยอะค่ะ

1.การใช้ชีวิตแบบเดิมนั้นเป็นยังไงครับ

2.ก็ไปไหนมาไหนไม่ต้องคอยกังวลว่าจะติดโควิดไหมค่ะ แต่ตอนนี้กังวลเยอะค่ะ

1.รู้สึกว่าการใส่แมสเป็นภาระไหมครับ หรืออาจะไมได้เป็นภาระแต่เราลืม ยังปรับตัวไม่ได้

2.ลืมค่ะ ยังปรับตัวไม่ได้ค่ะ

1.เคยเล่นกับเพื่อนกอดคอเฮอาระยะหลังเล่นไม่ได้

2.มีบ้างค่ะ

1.ปัจจุบันหนูมีการปรับตัวอย่างไรบ้างครับ

2.ปรับตัวได้หลายอย่างเลยค่ะ เช่น ทานข้าวก็ไม่ต้องนั่งใกล้กันขนาดนั้นค่ะ

1.เรื่องรักษาระยะห่างใช่ไหม แล้วมีอะไรบ้างเรื่องพฤติกรรม

2.ค่ะ ล้างมือใส่แมส เปลี่ยนไปเยอะเลยค่ะ

1.หนูเคยได้ยินเรื่องวัคซีนโควิดไหม แล้วได้ยินประมาณไหน

2.เคยได้ยินค่ะ ประมาณว่าวัคซีนไม่ค่อยมี ยังรักษาไม่ได้

1.พี่อยากรู้ว่าวัคซีนจะเป็นตัวช่วยป้องกันหรือยับยั้งโรคโควิดได้ดีที่สุด หนูเชื่อไหม คือปัจจุบันยังไม่มีวัคซีน

2.เชื่อค่ะ

1.ถ้าเขามีวัคซีนจะเอามาฉีดให้ประชาชน แล้วน้องเป็นคนหนึ่งที่จะได้ฉีดวัคซีน น้องจะไปฉีดไหมครับ

2.คิดดูก่อน

1.หมายความว่ายังไงครับ ถ้าหนูแข็งแรงหนูก็จะไม่ไปหรอครับ

2.ค่ะ เพราะร่างกายดีอยู่แล้วค่ะ

1.แล้วหนูคิดว่าจะไปฉีดตอนไหน อะไรถึงอยากทำให้ไปฉีดครับ

2.ตอนอยู่ในพื้นที่เสี่ยงแล้วก็เริ่มมีอาการค่ะ

1.ถ้าถามตอนนี้สถานการณ์ปัจจุบัน ถ้าในเลือก หนูคง....

2.ไม่ไปค่ะ

1.กลัวไหม กลัวอะไรในวัคซีนนั้นบ้าง

2.บางทีเราฉีดแล้วภูมิคุ้มกันเราอาจจะหายไปเลยก็ได้ค่ะ

1.แล้วหนูคาดหวังอะไรในตัววัคซีน

2.ถ้าฉีดแล้วก็ขอให้หายขาดเลยก็น่าจะดีค่ะ

1.กลัวหรือกังวลเกี่ยวกับวัคซีนไหม

2.มีบ้างห้าสิบห้าสิบ

1.ความมั่นใจในตัววัคซีน มั่นใจร้อยเปอร์เซ็นเลยไหมในตัววัคซีน

2.ไม่รู้อ่ะ

1.ที่ให้ห้าสิบห้าสิบนี้คือยังไงครับ

2.บางทีร่างกายเรามันดีอยู่แล้ว ฉีดอะไรเข้าไปมันอาจจะไม่เหมือนเดิมค่ะ ถ้าฉีดแล้วดีก็ดีแต่ถ้าฉีดแล้วไม่ดีก็คือการตัดสินใจผิดพลาดค่ะ

1.แล้วถ้าวัคซีนผลิตเรียบร้อยแล้ว แล้วจะมาฉีดให้หมู่บ้านเรา เขารณรงค์ว่าจะมาฉีดละ หนูจะมีส่วนร่วมในการมาช่วยรณรงค์ไหมครับ

2.หนูก็จะเฉยๆ

1.แล้วหนูจะบอกคนข้างบ้านไหมว่าจะมีการมาฉีดวัคซีนแล้ว

2.บอกอยู่แต่หนูอาจจะไม่ไปก็ได้ค่ะ บอกให้รู้ ไม่ได้บังคับ

1.อาจจะไม่ได้เชิญชวนให้ไปด้วยกัน ทำไมหรอครับ

2.ยาที่มาฉีดหนูก็ไม่รู้หนูยังไม่มั่นใจว่าวัคซีนมันดีหรือไม่ดี

1.อะไรที่ทำให้หนูไม่มั่นใจเกี่ยวกับวัคซีนหรือว่าหนูดูจากข่าว

2.ดูจากข่าวบ้างอะไรบ้าง

1.ข่าวที่หนูดูนี้ประมาณไหนครับ

2.รู้สึกว่าจะมีวัคซีนหลายตัวอยู่หรือป่าว

1.หรือดูข่าวว่าต่างประเทศฉีดแล้วก็ตาย

2.มีบ้างค่ะ

1.เคยอ่านในเฟสในไลน์เลยทำให้รู้สึกว่ามันยังไม่นั่งใช่ไหมครับ

2.เราไม่มั่นใจว่าฉีดเรามันจะหายหรือป่าวอะไรประมาณนี้ค่ะ

1.ยังไม่มีผลยืนยันว่าฉีดเข้าไปแล้วหายอะไรแบบนี้ใช่มั้ยครับ ถ้าเข้ามาจ้างเราเป็นหนูทดลองมาฉีดวัคซีนตัวนี้ได้ห้าพัน จะเอาไหมครับ

2.ไม่เอาค่ะ

1.ประสบการณ์ที่ผ่านมาของโควิด หนูคิดว่าโควิดนี้จะอยู่กับเราไปอีกนานไหมครับ

2.น่าจะหมดภายในปีนี้มั้ง เพราะว่าหลายคนมีการป้องกันตัวอยู่ค่ะ

1.หนูรู้สึกว่าคนเริ่มมีการรู้วิธีการป้องกันตัวมากขึ้น ก่อนหน้าที่จะมีโควิดส่วนตัวหนูได้มีการป้องกันและดูแลสุขภาพตัวเองอย่างไรบ้างครับ

2.ก็ใช้ชีวิตปกติ พอมีโควิดก็ป้องกันเยอะมากขึ้น ทำตามมาตรการ

1.ที่โรงเรียนเขามีการเข้มงวดไหม

2.เข้มงวดค่ะ ถ้าไม่มีแมสจะไม่ให้เข้าโรงเรียนค่ะ ล้างเจลแอลกอฮอร์ทุกครั้ง

1.แสดงว่าที่โรงเรียนก็อุ่นใจในระดับหนึ่ง แล้วพอกลับมาบ้านละ

2.ก็ล้างมือให้สะอาดค่ะ

1.ในชุมชนหนูได้มีส่วนร่วมในการป้องกันควบคุมโรคโควิดอะไรบ้างไหมครับ

2.ทำตามที่เชาบอกค่ะ

1.เขาบอกอะไรบ้าง

2.ก็ไม่ไปในที่คนเยอะๆนะใส่แมสค่ะ

1.ที่บอกว่าเขา เขานี้คือใครครับ

2.ผู้ใหญ่บ้านค่ะ

1.แล้วเขาบอกยังไงครับ ประกาศบอกหรืออะไรครับ

2.เขาประกาศเสียงตามสายค่ะ

1.หลักๆก็คืดใส่แมส ล้างมือ

2.ถ้าไม่จำเป็นก็ไม่ต้องออกไปไหนค่ะ

1.แล้วเราทำได้ไหมครับ

2.เล่นมือถือทุกวันๆก็เบื่อค่ะ ทำให้สายตาเสียด้วยค่ะ

1.โรงเรียนนี้เขาเปิดปกติไหมครับ

2.ปิด 3 เดือนเลยค่ะ ปกติ ปิด 2 ค่ะ

1.แล้วเราทำไง

2.อยู่บ้านแล้วก็เรียนออนไลน์ค่ะ ไม่เข้าใจเลย

1.เรียนออนไลน์เป็นไงบ้าง

2.ตอนนี้ไม่ค่อยมีเรียนออนไลน์

1.เรียนออนไลน์ที่ว่าไม่เข้าใจนี้ยังไง

2.บางทีเขาสอนไม่เหมือนคุณครู

1.แล้วใครมาสอนครับ

2.ไม่ใช่อันที่เขาสอนเราเลยไม่เข้าใจ

1.แล้วเพื่อนเป็นยังไงบางคนมีอินเทอร์เน็ตไม่มีอินเทอร์เน็ต แล้วเขาสอนกันยังไง

2.บางคนก็ไม่ค่ะ

1.ถ้าเลือกได้ก็อยากเรียนในห้อง ห้องเรียนดีสุดแล้วใช่ไหมครับ

2.ในห้องเรียนดีสุดค่ะ

1.ความคาดหวังสถานการณ์นี้ถือว่าดีบ้างไม่มีบ้าง หนูคาดหวังอะไรจากภาครัฐที่จะให้ภาครัฐช่วยอะไรในสถานการณ์โควิดนี้ครับ

2.ช่วยกันป้องกัน จะได้หายเร็วๆ

1.แล้วจะให้รัฐป้องกันยังไงครับ

2.ปิดประเทศไปเลย ไม่ให้คนนอกเข้ามา เพราะว่าการที่คนนอกไปมาทำให้เชื้อไวรัสมาติดได้

1.มาตรการเยี่ยวยาอยากให้เขาช่วยอะไรไหม ด้านเศรษฐกิจ สังคม

2.เรื่องเงิน เพราะเศรษฐกิจไม่ค่อยดี

1.อยากให้เขาทำประมาณไหน

2.จ้างงานก็ได้ค่ะ เพราะ ช่วงนี้ไม่มีงาน

1.ความคาดหวังต่อบุคลากรทางการแพทย์ละหมอ พยาบาล หมออนามัย คาดหวังอยากให้เขาช่วยอะไรหนูบ้าง

2.ด้านบริการค่ะ บริการดี

1.บริการดีหมายถึงตอนนี้เขาบริการไม่ค่อยดีหรอครับ

2.ก็มีบ้างค่ะ บางคน

1.แล้วช่วงสถานการณ์โควิดอยากให้เขาเข้ามาช่วยอะไรบ้าง มาตรวจมาอะไรแบบนี้

2.ออกมาตรวจสุขภาพเลย ถ้าเราไปตรวจเองมันแพง บางคนไม่มีตังก็ไม่ได้ตรวจ

1.ความคาดหวังต่อคนในหมู่บ้านคาดหวังว่าอยากให้เป็นยังไงครับ

2.ไม่น่าจะป้องกันอะไรได้มากค่ะ ก็ใส่แมสป้องกัน รักษาความสะอาด

1.คนบ้านเราปฏิบัติตามไหมครับ

2.ก็ทำช่วงแรกๆ แต่ตอนนี้ไม่ค่อยเท่าไร ปฏิบัติ 40 เปอร์เซ็นต์

1.แสดงว่าคนไม่ปฏิบัติมากกว่า น้องคิดว่าเพราะอะไรครับ

2.ก็คคิดว่าไม่ติดหรอกมั้ง ยังไงก็ไม่มาถึงเราหรอก

1.ทำไมถึงคิดว่าไม่มึงเราหรอก

2.เพราะว่าชุมชนของเราคนไม่เยอะเท่าแม่สาย

1.บ้านเรามีคนมาจากพื้นที่เสี่ยงไหม

2.มีค่ะ แต่ก็กักตัวไปแล้วค่ะ

1.ในชุมชนเรามีระบบการกักตัวอย่างไรบ้างครับ

2.กักตัว 14 วันค่ะ ไปกักตัวที่วัดค่ะ หลังวัดค่ะมีที่กว้าง

1.มีที่เดียวหรอครับ ใช้กี่หมู่บ้านครับ

2.มี สองที่วัดกาคำกับวัดกาข้าว

1.โควิดทำให้หนูได้เรียนรู้อะไรบ้างครับ

2.หลายอย่างเลยค่ะ ได้เรียนรู้ความน่ากลัวของมัน การป้องกันต่างๆ การปรับตัวมีบ้างค่ะ

1.ผ่านไปปีกว่าแล้ว หนูคิดว่าหนูได้บทเรียนอะไรจากโควิด

2.บทเรียนได้การวิตกวังวลบ้าง

1.หนูคิดว่าตัวเองมีความเข้มแข็งขึ้นไหมในถานการณ์โควิด

2.คิดไม่ออกค่ะ

1.แต่อย่างน้อยก็รู้ว่าโรคนี้น่ากลัวส่งผลกระทบนะ

2.ทางด้านการเงินต่างๆค่ะ

1.ผู้สัมภาษณ์ 2.ผู้ถูกสัมภาษณ์

**P5อาเหมย.m4a**

1.พี่อาเหมยแต่งงานหรือยังครับ

2.แต่งแล้วค่ะ

1.มีลูกกี่คนครับ

2. 2คนคะ ผู้หญิงหนึ่งผู้ชายหนึ่ง ไปทำงานกันหมดแล้วค่ะ

1.ลูกอายุเท่าไหร่ครับ แล้วอยู่ที่ไหน

2. ผู้ชายอายุ 21 ปีค่ะ อยู่กรุงเทพ ผู้หญิง 20 ค่ะ อยู่กรุงเทพ

1.ลูกไปอยู่กรุงเทพกี่ปีแล้วครับ

2. 5 ปีแล้วค่ะ

1.ไปทำงานอะไรครับ

2.ผู้หญิงไปช่วยเถ้าแก่คิดบันชี ผู้ชายเป็นช่างเฟอร์นิเจอร์

1.ช่วงโควิดลูกไม่ได้กลับมาเลย ไม่ให้กลับหรืออย่างไรครับ

2.ไม่ให้กลับค่ะ กลัวโควิดค่ะ

1.คิดถึงก็โทรหากันเอา ทุกวันนี้อยู่กับใคร

2.อยู่กับแฟน

1.ทำงานอะไรครับ

2.รับจ้างทั่วไป ทำความสะอาดบ้านค่ะ ได้เป็นรายวัน วันละ 300

1.แฟนพี่ทำงานอะไรครับ

2.ช่างประปาค่ะ

1.เงินเดือนพอใช้ไหมครับ

2.ไม่พอใช้ค่ะ

1.แล้วลูกส่งมาให้ใช้บ้างไหมครับ

2.ถ้ามีก็ส่ง ไม่มีก็ไม่ส่ง

1.แสดงว่าตอนที่มีโควิดทำให้รายได้เราลดลงไหมครับ

2.ของพี่ก็ลดลงและของแฟนก็ลดลง

1.มันลดลงยังไงครับ

2.งานก็ลดลงรายได้ก็ลดลง

1.ช่วงโควิดที่ผ่านมา สุขภาพเป็นยังไงบ้างครับ

2.ยังแข็งแรงเหมือนเดิมค่ะ จิตใจก็ไม่มีความวิตกกังวลอะไรค่ะ

1.ในช่วงโควิดที่ผ่านมาที่เขาประกาศห้ามชุมนุม ส่งผลกระทบต่อด้านสังคมเราไหม เช่นเราจะไปวัด ทำกิจกรรม ขึ้นบ้านใหม่ ได้รับผลกระทบไหมครับ

2.ลำบากค่ะ

1.แล้ววิถีชีวิตได้เปลี่ยนไปไหมครับ

2.เปลี่ยนค่ะ กลัวโควิด ทำงานไม่ได้

1.ในความคิดของพี่โรคโควิดคือโรคอะไรในความเข้าใจพี่

2.เป็นโรคที่น่ากลัว

1.ติดต่อได้ยังไง

2.ระบบหายใจค่ะ

1.เคยรู้สึกกังวลว่าเราจะติดโควิดไหมครับ

2.กลัวค่ะ อยู่ด้วยกันก็กลัวค่ะ ต่างคนต่างกลัวค่ะ

1.ใครคือกลุ่มเสี่ยงที่จะเป็นโรคโควิดครับ

2.กรุงเทพค่ะ เมืองจีน เมืองนอกค่ะ

1.ตัวพี่เองคิดว่าเสี่ยงไหม เสี่ยงยังไง

2.เสี่ยงเหมือนกัน เพราะเมืองนอกเข้ามาอยู่

1.มาไหนมาในชุมชนเราหรอนักท่องเที่ยวหรอครับ

2.นักท่องเที่ยวค่ะ เขาขโมยเข้ามาอยู่ก็มีค่ะ

1.ความรุนแรงโรคโควิดน่ากลัวรุนแรงไหม เป็นแล้วรักษาหายไป

2.น่ากลัวมากค่ะ รุนแรงมากค่ะ

1.โรคโควิดน่ากลัวเทียบเท่าโรคอะไรในความคิดของพี่

2.โรคโควิดมาที่สุด

1.การป้องกันตัวโควิดมีอะไรบ้างที่พี่รู้มา

2.ล้างมือ ใส่หน้ากากทุกวันเลยค่ะ อย่าเอามือไปโดนตา ออกไปข้างนอกกลับมาต้องอาบน้ำ

1.แล้วมันทำยากไหมครับ

2.ไม่ยากค่ะ ทำจนชินแล้วค่ะ

1.เพิ่งจะมาทำช่วงที่มีโควิดหรือทำมานานแล้วครับ

2.ทำในช่วงโควิด ทำจนชินแล้วค่ะ

1.เคยออกไปข้างนอกแล้วลืมหน้ากากไหมครับ

2.จำได้อยู่ค่ะ กลับมาต้องล้างมือค่ะ เคยชินแล้ว ตอนแรกชอบลืมแมส

1.ตอนนี้ต้องอยู่กับอันนี้มาไม่ลืม เอาไว้ที่ไหนบ้าง

2.เอาแขวนไว้มอไซต์ เก็บไว้ในกระเป๋า ต้องใส่สองสามที่นะ

1.วัคซีน เคยได้ยินวัคซีนป้องกันโรควิดไหม

2.เคยได้ยินอยู่ค่ะ

1.ได้ยินว่ายังไงบ้าง

2.ได้ยินมาว่าเมืองไทยยังไม่มี มีที่เมืองนอก ลืมไปแล้วว่าประเทศอะไร ต้องผลิตก่อน

1.ได้ยินข่าวมาจากไหนเรื่องวัคซีน

2.มือถือ กับทีวี

1.ตอนที่ได้ยินเกี่ยวหับวัคซีนที่มีข่าวว่าฉีดวัคซีนแล้วตายไหมครับ

2.ไม่เคยได้ยินค่ะ คนตายก็ไม่กล้าไปกลัวโควิด งานแต่งก็ไม่กล้าไป

1.ถ้ามีวัคซีนมาให้พี่ฉีดไหม

2.ถ้าคุณหมอเรียกฉีดก็ฉีด คนอื่นก็ยังฉีดได้เลย พี่ก็ฉีดได้เหมือนกัน กลัวแต่ว่าฉีดเข้าไปแล้วมันไม่ถูกต้องก็มี กลัวแพ้เหมือนกัน ไม่ฉีดก็ไม่ได้

1.กลัวผลข้างเคียงเหมือนกัน พี่คิดว่าพี่จะมีโอกาสได้รับวัคซีนนั้นไหมครับ

2.ได้ค่ะ

1.ทำไมถึงคิดว่าตัวเองถึงจะได้รับโอกาสฉีดวัคซีนนั้น

2.เพราะโควิดน่ากลัว ต้องอยากฉีดวัคซีนนั้น

1.ถ้ามีการฉีดวัคซีนจะเชิญชวนให้ข้างบ้านไปฉีดวัคซีนไหม

2.ถ้ามีชื่อเขาก็ต้องเรียกเขาไปฉีด ถ้าไม่มีก็ไม่เรียกค่ะ

1.พี่เป็น อสม ไหม เขาให้ทำอะไรบ้าง

2.เป็นอยู่ค่ะ บอกให้ชาวบ้านใส่หน้ากาก บอกให้ล้างมือค่ะ

1.เป็น อสม มากี่ปีแล้ว ได้เงินไหมครับ

2.เป็นมา 8 ปีแล้วค่ะ ได้ค่ะ

1.ไหนบอกบทบาท อสม หน่อยครับว่าต้องทำอะไรบ้างครับ

2.ไปตรวจคนไข้ค่ะ พี่ดูแล 10 หลังค่ะ แล้วก็ไข้เลือดออกยุงลายค่ะ ไปแจกหน้ากากอนามัยค่ะ เจลล้างมือค่ะ

1.เท่าที่สังเกตุดูคนในพื้นที่ที่พี่ดูแลเขาให้ความร่วมมือไหมครับ

2.ให้ความร่วมมือค่ะ อสม พูดยังไงเขาก็ทำตามเลยค่ะ ไม่ขัดไม่ขืนเลยค่ะ

1.มีใครที่ไม่ทำตามไหนครับ

2.ไม่ค่ะ ในพื้นที่พี่ไม่มีค่ะ

1.แล้วของคนอื่นละครับ

2.ของคนอื่นพี่ก็ไม่รู้ค่ะ

1.ในชุมชนของเรามีกลุ่มเสี่ยงมาไหม

2.เขากลับมาจากกรุงเทพแล้ว อสม ก็ไปตรวจค่ะ ทุกวันค่ะ

1.เขาอยู่บ้านเขาหรอครับ แล้วอายุช่วงเท่าไหร่

2.เขาอยู่บ้านเขาค่ะ ไม่ออกมาเลย ช่วงวัยรุ่นไปทำงานกลับมากักคัวค่ะ อสม จะไม่ให้ออกมาเลยค่ะ ให้อยู่บ้านเลย

1.กักตัว 14 ครับ แล้วถ้ามีการรณรงค์พี่ก็ต้องไปช่วยใช่ไหมครับ

2.ช่วยค่ะ คุณหมอน่าจะสั่ง

1.หมออนามัยหรือหมอโรงพยาบาลครับ

2.หมออนามัยค่ะ หมอให้ช่วยก็ต้องช่วยเขาค่ะ อสม เขาไม่รู้ค่ะถ้าไม่สั่ง

1.ในหมู่บ้านเรามี อสม กี่ คน ครับ

2. 40 คนค่ะ หมู่บ้านเราหมู่บ้านเดียวค่ะ

1.วัคซีนที่จะฉีดเกี่ยวกับโรคโควิดเนี้ย พี่คาดหวังอะไรในตัววัคซีนครับ

2.ป้องกันโควิดและก็ไข้หวัดใหญ่ไข้เลือดออก ให้ป้องกันโรคได้เลย

1.พอมีโควิดเข้ามาพี่ต้องรับผิดชอบอะไรเพิ่มจากที่หมอสั่งไหมครับ

2.ก็ต้องไป หมอสั่งมาก็ต้องไปค่ะ

1.เวลาที่พี่รายงานว่าถ้าหมู่บ้านเรามีกลุ่มเสี่ยงมา พี่ต้องไปแจ้งใครครับ

2.แจ้งที่คุณหมอแล้วก็ผู้ใหญ่บ้าน ถ้าไม่มีเวลาก็โทรไป ถ้ามีเวลาก็ไปหา

1.พี่คาดหวังอยากให้รัฐบาลช่วยเหลืออะไรบ้างครับ

2.ช่วยเหลือความเป็นอยู่ เรื่องงานเรื่องเศรษฐกิจไม่เอาค่ะ

1.แล้วอยากให้เจ้าหน้าที่บุคคลากรทางการแพทย์ช่วยเหลืออะไรบ้างครับ

2.ช่วยเหลือหน้ากากแล้วก็เจลล้างมือ

1.อยากให้ผู้ใหญ่บ้านช่วยเหลืออย่างไรบ้างไหมครับ

2.ช่วยใส่หน้ากากอนามัย ล้างมือ ปฏิบัติตามที่เขาแนะนำ ต้องอยู่กับเพื่อนเป็นกลุ่มไม่ดี

1.พี่ได้เรียนรู้อะไรจากโควิดที่ผ่านมา

2.ได้เรียนรู้ว่าต้องใส่หน้ากากปิดปาดให้ดีๆ เวลากินข้าวกินอาหารก็ต้องล้างมือให้สะอาด ไม่ให้อยู่เป็นกลุ่มๆ

1.พี่ว่าโควิดจะอยู่กับเราอีกนานไหม

2.อยากให้หายเร็วๆ ไม่รู้จะหมดไม่หมด น่าจะอีก 2 ปี

1.ก่อนที่รอบที่สองจะมาเชียงราย พี่คิดว่ามันจะหายไปหรือยังในช่วงตุลาพฤศจิกา

2.คิดว่าจะหาย แต่มาอีกจากฝั่งพม่า ข้ามมาจากฝั่งโน้น

1.เหนื่อยที่ต้องดูแลตัวเองไหม

2.ดูแลคนอื่นเหนื่อยนะ ดูแลตัวเองไม่เหนื่อยเพราะเราทำจนชินแล้ว

1.ผู้สัมภาษณ์ 2.ผู้ถูกสัมภาษณ์

**P7วลีรัตน์.m4a**

1.แต่งงานหรือยังครับ

2.แต่งแล้วก็เลิกค่ะ เลี้ยงลูก

1.มีลูกกี่คนแล้วอายุเท่าไหร่ครับ

2. 3 คน ค่ะ คนโตผู้หญิง 9 ขวบ สองผู้ชาย 6 ขวบ คนสุดท้าย ผู้ชายขวบแปดเดือนค่ะ

1.ขอโทษนะครับทั้งสามคนคุณพ่อเดียวกันไหมครับ

2.ค่ะ เพิ่งมาเลิกช่วงโควิดนี้ค่ะ ตรุษจีนนี้ก็ครบ 1 ปี พอ ตั้งแต่เลิกกันก็ไม่ส่งเสียอะไรเลย เขาไปมีใหม่เป็นคนเทอดไทยค่ะ

1.ทุกวันนี้อาชีพหลักของพี่คืออะไรครับ

2.ไม่มีค่ะ ไม่ได้ประกอบอาชีพ

1.เหตุผลที่ไม่ได้ประกอบอาชีพคืออะไรครับ

2.ไม่มีเวลาต้องดูแลลูก ดูแลพ่อแม่ค่ะ รับส่งลูกเรียนทำนู้นทำนี้ มีพี่สาวคนโตส่งมาให้ใช้ แต่ไม่พอใช้ค่ะ

1.ช่วงโควิดเนี้ยสิ่งที่พี่ได้รับผลกระทบแน่ๆทางด้านเศรษฐกิจนะครับ เงินไม่พอใช้นะครับ ทั้งดูแลรับผิดชอบครอบครัวตรงนี้เป็นหลักทั้งหมดเลยนะครับ ด้านสุขภาพร่างกายมีผลกระทบไหมครับ สุขภาพทางด้านจิตใจละครับ

2.ทางด้านร่างกายไม่มีค่ะ ทางด้านจิตใจก็มีคิดมาก

1.คิดมากนี้ยังไงจากด้านโควิดหรอครับ

2.มันก็หลายอย่าง โควิดด้วย เรื่องลูกด้วย เรื่องแฟนด้วย แต่หลักๆคือเรื่องลูก

1.ลูกนี้เรากังวลเรื่องอะไรบ้างครับ

2.เรื่องค่าใช้จ่าย เพราะแฟนก็ไม่ส่งเสียอะไรเลย

1.แล้วแฟนเขามีลูกใหม่ไหมครับ

2.มี 1 คน อยู่ใกล้กันด้วย

1.มันก็มีเรื่องกวนใจนะครับทั้งเรื่องงานเรื่องแฟนไหนจะเรื่องโควิด ออยู่ใกล้กันด้วย

2.แล้วอยู่ใกล้แค่นี้ลูกก็ไม่มาดูแล

1.มาทางด้านโควิด ทำให้การใช้ชีวิตพี่เปลี่ยนไปไหมครับ ตั้งแต่มีโควิด

2.ก็เปลี่ยนไปหลายอย่างทั้งการเดินทางไปไหน ก็จะระแวงกลัวว่าจะติดโควิด

1.คิดเหมือนกันใช่ไหมว่าจะติดโควิด ไหนจะเดินทางเข้าไปในเมือง

2.ใช่ ถ้าเราไม่ไปก็ไม่ได้ ต้องไปซื้อของ แต่เราต้องป้องกันตัวเอง

1.การเดินทางเราก็ต้องระมัดระวังตัวเองมากขึ้น

2.ใช่ๆ ยิ่งเวลาลูกไม่สบายไปข้างล่างลำบากมาเลย ขับมอไซต์ไปอันตราย คลินิกจะเปิด 5 โมงเราต้องลงไปเอาลูกแบกไปข้างหลัง มันลำบากจริงๆเวลาลูกไม่สบาย

1.โรคโควิด พี่เข้าใจว่าโรคโควิดเกิดจากอะไรครับ

2.เกิดจากสิ่งแวดล้อม หายใจ อากาศ พี่ว่าเป็นโรคติดต่อทางเดินหายใจ

1.พี่พูดเองว่าก็มีกังวลในการเดินทางไปไหน

2.ใช่ค่ะ เพราะว่ามีแต่พี่คนเดียวที่ไปตลอด ถ้าลูกจะไปหาหมอ ไปนู้นนี้ แม่เชียงราย ก็เป็นพี่ตัวเราเองไปคนเดียว อย่างแม่คราวก่อนก็ไปเฝ้าและออกมีโอกาสสัมผัสคนนั้นคนนี้ กลัวเอาไปติดลูกอีก เราคิดแบบนั้นปุ๊บ เราจะไม่สบายใจเลย เพราะเราตัวคนเดียวที่ต้องวิ่งดู เหมือนเสาหลักของบ้านเลยวิ่งไปรับลูก ช่วงที่ขายน้ำเต้าหู้ไปขายของ พี่สาวกับพี่เขยทำน้ำเต้าหู้บ่ายสามออกไปขายของกลับมา 1 ทุ่ม ไม่ไปก็ไม่ได้ลูกไม่มีกิน โดนแฟนทิ้งอีกเลย โควิดมานี้นะงานก็ไม่มีทำ แต่ยังดีที่พี่คิดได้ คิดได้ว่าไม่ต้องคิดมากอะไรที่ผ่านก็ให้ผ่านไป อะไรจะเกิดก็ให้มันเกิด แต่เราต้องอยู่ต้องทนเพื่อลูกและครอบครัว

1.พี่เป็นคนแบบนี้มาตั้งแต่สมัยสาวๆอยู่แล้วไหมครับ เป็นคนที่ไม่คิดอะไรมาก

2.ไม่ใช่นะ เป็นคนคิดมาก คิดมากเป็นอันดับแรก แล้วเป็นคนระแวง

1.ตอนนี้ก็รู้แล้วว่าคิดมากไปก็ไม่มีอะไรดีขึ้น

2.คิดมากไปก็ไม่ได้อะไรคิดบุ๊ปได้ปั๊บแบบนั้นก็ได้ แต่พอเราคิดมาก เราก็ได้แต่โรคติดตัวเลยปล่อยวาง ถ้าเราคิดมากร้องไห้แล้วลูกจะทำยังไง ถ้าพี่ทำตัวเหมือนแฟนไปเลย แล้วลูกจะอยู่ยังไง ลูกจะเอาเสาร์หลักที่ไหนยืนเกาะ

1.เราก็คิดไปอีกแบบหนึ่ง เราก็จ้องเข้มแข็งไปอีกแบบ เยี่ยมมากเลยนะครับ แล้วโอกาสเสี่ยงพี่คิดว่าตัวพี่เองเป็นกลุ่มเยงไหมครับ

2.ก็ถ้าเราไม่ออกไปไหนอยู่บ้าน ก็ไม่เป็นไร มันอยู่ที่ภูมิต้านทานของเราด้วย ถ้าเราแข็งแรงมันก็ไม่เป็นอะไร ถามว่าเสี่ยงไหมก็เสี่ยงจากคนข้างนอกกลับมา เขาจะมีหรือไม่มีก็ไม่รู้ต่างคนต่างกลัวกัน แต่เราก็ต้องป้องกันเป็นอันดับแรก เพราะเราต้องไปเจอคนนั้นคนนี้

1.ในความคิดของพี่ทุกวันนี้ใครคือกลุ่มเสี่ยงของโรคนี้

2.มาจากเมืองนอก ประเทศจีน คนที่อยู่ในเมือง กรุงเทพแบบนี้ เชียงใหม่ คนที่มาจากพื้นที่เสียง

1.พี่คิดว่าโรคนี้มันรุนแรงมากไหมครับ

2.รุนแรง ติดก็คือตายเลย เสียชีวิตเลย คิดว่าจะรักษาหายไหม ไม่น่าจะหาย แต่ถ้ามียาก็น่าจะบรรเทาได้

1.ถ้าเปรียบเทียบกับโรคต่างๆโควิดนี้มันพอๆกับโรคอะไรครับ

2.พอๆกับไข้เลือดออก ไข้หวัดใหม่ เวลาไข้เลือดไข้ขึ้นสูงจะสูงมาก พี่เคยไปเฝ้าแม่

1.วิธีการป้องกันพี่น่าจะทราบอยู่แล้วนะครับ ไหนลองสรุปให้ฟังอีกทีครับ

2.ทราบค่ะ ผ้าปิดปากตลอด มีไว้ในมอไซต์ มีไว้ในกระเป๋าตลอด เวลากลับมาบ้านก็ล้างมือล้างเท้า ใช้เลล้างมือค่ะ

1. พี่ถือว่าทำได้ปกติไม่มีปัญหานะครับ

2.ค่ะ แต่บางครั้งก็ลืมไปเซเว่นลืมก็ซื้อเพิ่ม

1.ของพี่เนี้ยทำจนชินแล้วโอเคทำได้ แล้วพี่คิดว่าคนในชุมชนหมู่บ้านเราเท่าที่พี่สังเกต เขาให้ความร่วมมือดีไหมครับ

2.ในความรู้สึกพี่บางคนเขาก็ใส่ บางคนก็ไม่ใส่ ก็มีแต่ส่วน บางทีเขาก็ลืมแล้วไปซื้อร้านค้า แต่ส่วนมากจะใส่

1.สัดส่วนคนในชุมชนที่ให้ความร่วมมือกับคนที่ไม่ให้ความร่วมมือ อันไหนมากกว่ากัน

2.คนให้ความร่วมมือมากกว่า ประมาณ หกสิบเปอร์เซ้น

1.แล้วพฤติกรรมล้างมือนี้ครับ พี่คิดว่าตัวพี่เองทำได้ตลอดไหมครับ

2.ตลอดอยู่แล้วค่ะเพราะพี่ต้องทำทุกอย่าง

1.แล้วส่งผลไปถึงลูกถึงแม่พี่ไหมครับ

2.พี่ดูแลให้ตลอดหมดเลย ดูแลครอบคลุมพี่เป็นคนดูแลให้หมดเลย พ่อกับแม่ไม่ค่อยได้ออกไปไหนค่ะ

1.แล้วเรื่องวัคซีนละครับ พี่เคยได้ยินเรื่องวัคซีนโควิดไหมครับ เคยได้ยินจากไหน

2.เคยได้ยินค่ะ จากในทีวี

1.ได้ยินมาว่ายังไงครับ

2.ได้ยินมาว่าจะมียาวัคซีน

1.แล้วตอนนี้มียังในประเทศไทย จากที่พี่ยินมา

2.ยังค่ะ ยินว่าจะเขาแต่ไม่ใช่ว่าจะรักษาได้หาย แต่มันบรรเทาได้ค่ะ ไม่ใช่ฉีดปุ๊บจะหายนะคะ

1.พี่คิดว่าถ้ามีวัคซีนพี่จะฉีดไหมครับ

2.ฉีดค่ะ เพราะป้องกันโควิด

1.พี่ไม่กลัวผลกระทบที่จะทำให้เกิดการไม่สบายเกิดขึ้นหรอครับ

2.ก็คิด แต่ถ้าคนอื่นฉัน เราก็กล้าฉีด หมอให้ฉีดเราก็ฉีด เพราะถ้าเราเป็นอะไรหมอเป็นคนดูแล

1.ก็คือตามคำสั่งหมอถ้าหมอให้ฉีดก็ฉีดนั้นแหละ

2.เพราะหมอดูแลเราอยู่แล้ว

1.พี่เชื่อว่าวัคซีนจะมีประสิทธิภาพดีไหมครับ

2.ดีแต่ว่าจะไม่หายหมด แต่ก็แค่บรรเทา แต่ถ้าจะให้หายจากโรคนี้ไปเลยคงจะเป็นไปไม่ได้

1.พี่คาดหวังว่าอยากให้วัคซีนที่ฉีดนี้เป็นยังไง ฉีดแล้วให้เป็นยังไง

2.อยากให้ครอบคลุมโรค ป้องกันโรค

1.ถ้าเขามีการรณรงค์ฉ๊ดวัคซีนพี่จะเข้าร่วมไหม

2.ร่วมค่ะ

1.ด้วยวิธีการใดครับ

2.ช่วยเขาบอกต่อ ให้ฉีดวัคซีนนะ

1.พี่คาดหวังว่าภาครัฐช่วยเหลืออะไรบ้างในสถานการณ์โควิด

2.อยากให้ช่วยทุกอย่าง ของกิน อย่างพี่นี้กระทบ อยากให้ช่วยหมดเลย การเรียนของลูก มันต้องใช้ค่าเทอม

1.อยากให้หมอที่โรงพยาบาลช่วยเหลืออะไรครับ

2.อยากให้เขามาตรวจเช็คสุขภาพ บางทีพี่ไม่อยู่บ้านก็ต้องให้เขามีหมอมาตรวจตามบ้าน ลูกๆไม่อยู่ คนแก่ไปไม่ได้อยู่แล้ว

1.แล้วคนในชุมชนละครับ อยากให้เขาทำอย่างไรในสถานการณ์โควิดแบบนี้

2.อยากให้เขาช่วยเหลือดูแลซึ่งกันและกันให้เขาปิดจมูก ปิดปากไว้ ไม่อยากให้เขาอยู่เป็นกลุ่ม อยากให้มีสถานที่กักตัวยาก ไม่อยากให้เขากลับมากักตัวที่บ้าน แยกออกไปต่างหาก ถ้ากักตัวที่บ้านแล้วคนที่อยู่ในบ้านละ ก็ติดกันหมด เหมือนมีส่วนกลาง ถ้ากักตัวที่บ้านอีกคนหนึ่งติด สี่คนไม่ติด จะทำยังไง มันก็วุ่นวายกันไปหมด

1.แสดงว่าที่บ้านเราสถานที่กักตัวส่วนกลางยังไม่มี

2.ยังไม่มีค่ะ บ้านใครบ้านมัน

1.เมื่อวานผมไปเทิดไทยเขามีหลังวัดไว้กักตัว แบ่งไว้ชัดเจน ไม่ใช่กักที่บ้านใครบ้านมัน แล้วจากประการณ์ที่ผ่านมาละครับ ก่อนจะมีโควิดพี่ก็ดูแลแบบนี้เหมือนเดิมหรอครับ

2.ใช่ เพียงแต่ว่าแมสไม่ได้ใส่ แต่โควิดมาก็บ่อยขึ้น ไปไหนก็ติดแมสตลอดค่ะ

1.ระหว่างการระบาดก็คือดูแลแบบเต็มที่

2.ตัวพี่ไม่เป็นไร แต่ตัวลูกพี่พ่อแม่พี่อย่าเป็นอะไร สำคัญที่สุดเลย ถ้าเขาเป็นอะไร ก็จะมีแค่พี่คนเดียวที่ต้องไปเฝ้า พี่ก็เลยต้องทำอะไรก็ได้ให้เขาแข็งแรงเข้มแข็งที่สุด ถ้าพี่เป็นไรก็คือทำไรไม่ได้

1.ท้ายที่สุดโควิดในช่วงที่ผ่านมาเราได้บทเรียนอะไรบ้างในสถานการณ์โควิด

2.บทเรียนหรอมันก็สอนอยู่นะ พยายามไม่ออกไปข้างนอก ไม่พยายามรวมกลุ่มกัน เพราะมันดีต่อเรา ไม่ออกไปข้างนอก แต่ไม่ออกไม่ได้ เราจำเป็น

1.แล้วสถานการณ์โควิดทำให้เราปล่อยวางอะไรได้มากขึ้นไหมครับ หรือว่าไม่เกี่ยว

2.ถ้าถามว่าเกี่ยวไหม พี่ก็ไม่รู้ไม่เคยมาเจอแบบนี้ ในเมื่อมาเจอแล้ว เราก็ต้องตั้งไว้ แต่ก็เป็นหนีเพิ่ม เพราะแฟนคนเดียวทำให้พี่เป็นหนี้เพิ่ม จากเงินรัฐ 40000 อันนั้นเป็นชื่อแฟนแต่พี่ต้องรับผิดชอบหมดเลย แต่พี่ไม่ไปยุ่งกับเขาแต่อย่ามายุ่งกับลูกพี่ แต่ถึงยังไงเขาก็ยังเป็นพ่อของเด็ก พูดยากไงเรื่องครอบครัว คิดมากไปก็ไม่มีประโยชน์ไง ในเมือเขาไม่หาเลี้ยงก็ช่างเขา มีคนหลายคนที่ต้องเหตุการณ์นี้
