# Supplementary material for: Impacts of and survival adaptations to the COVID-19 pandemic among the hill tribe population of northern Thailand: A qualitative study
Source: PLoS One. 2021 Jun 4;16(6):e0252326. doi: 10.1371/journal.pone.0252326 (PMC8177523; doi:10.1371/journal.pone.0252326)
Supplement: Attachment — Submitted filename: Response to reviewers comments.docx [file pone.0252326.s004.docx]

Response to editor and reviewer’s comments

Dear Editor,

Thank you very much for all valuable comments and suggestions. We have revised, and improved all points of concerns. We changed the word of “epidemic” to “pandemic”, improved all section of methods using the COREQ as guideline, added table 1 to present the general characteristics of participants, added a brieft introduction on ech section

Thank you for submitting your manuscript to PLOS ONE. After careful consideration, we feel that it has merit but does not fully meet PLOS ONE’s publication criteria as it currently stands. Therefore, we invite you to submit a revised version of the manuscript that addresses the points raised during the review process.

This is in interesting manuscript about a population that has not received much attention amid the pandemic. Strengths are that it is largely well written, there is a reasonable amount of data presented in the quotations to support the themes, and the overview and synthesis is good. And I appreciate the focus on adaptation and resilience. However, the following will help the manuscript be acceptable for publication:

: Thank you so much

The methods require more detail. In particular, more specific information is needed on participant recruitment, data collection, and data analysis (e.g., who analyzed? what methods? citations for the data analytic method are needed; how did you deal with discrepancies in the coding?). PLOS One requests that you complete and attach the COREQ checklist to identify important aspects of the methods; in fact this will assist with identifying the missing information in the Methods section; some of the points in the checklist should be described in the text of the manuscript. (Note that it is not expected that researchers will have taken all the steps or have all the information requested in the COREQ checklist; however, many of these items should be addressed. Please see other qualitative articles in PLOS One for guidance on how they have used COREQ.)

: Thank you so much for the suggestion in use the COREQ as the guideline to explain the method section, it really helps in extending the context of method section.

: We have put a lot of the information in the method section including interviewers’ background and experience, relationship development between interviewers and interviewees, methodological orientation, method of data collection, setting, data collection and data analysis, please revised detail in page 4, lines 120-124, and 130-134; page 5, lines 136-150.

In addition to the brief introductions to each section, rather than only presenting lists of quotation, please provide at least some brief overview statement at the end of each section or some explanation of at least some of the quotations integrated with the text. It is not sufficient to merely list the quotations in each section; this presumes the reader should do the work of interpreting them and linking them to the themes. It also might be helpful to provide 1 table with the participant number, gender, and age (and perhaps other relevant information if you have it). Finally, as reviewer 1 indicates, please provide brief recommendations based on your findings; understandably these should be tentative based on 1 qualitative study.

:Thank you for the great comment

: We have added brief introduction and summary at the end of each section, please see page 7, lines 175-177, and 194-195; page 8, lines 200-204; page 9, lines 246-247, and 254-256; page 10, lines 274-275 and 285-288; page 11, lines 304-305, 312-313, and 327-329; page 12, lines 344-346, 352-354, and 360-361; and page 13, lines 381-382; page 14, lines 403-404, 408-410 and 425-426.

: We have added table 1 to present the characteristic of participants, please see in page 5-6.

: we have added recommendations in the conclusion sections in page 18, lines 537-543.

Please submit your revised manuscript by May 10 2021 11:59PM. If you will need more time than this to complete your revisions, please reply to this message or contact the journal office at [plosone@plos.org](mailto:plosone@plos.org). When you're ready to submit your revision, log on to <https://www.editorialmanager.com/pone/> and select the 'Submissions Needing Revision' folder to locate your manuscript file.

Please include the following items when submitting your revised manuscript:

- A rebuttal letter that responds to each point raised by the academic editor and reviewer(s). You should upload this letter as a separate file labeled 'Response to Reviewers'.
- A marked-up copy of your manuscript that highlights changes made to the original version. You should upload this as a separate file labeled 'Revised Manuscript with Track Changes'.
- An unmarked version of your revised paper without tracked changes. You should upload this as a separate file labeled 'Manuscript'.

If you would like to make changes to your financial disclosure, please include your updated statement in your cover letter. Guidelines for resubmitting your figure files are available below the reviewer comments at the end of this letter.

If applicable, we recommend that you deposit your laboratory protocols in [protocols.io](http://protocols.io) to enhance the reproducibility of your results. Protocols.io assigns your protocol its own identifier (DOI) so that it can be cited independently in the future. For instructions see: <http://journals.plos.org/plosone/s/submission-guidelines#loc-laboratory-protocols>. Additionally, PLOS ONE offers an option for publishing peer-reviewed Lab Protocol articles, which describe protocols hosted on [protocols.io](http://protocols.io). Read more information on sharing protocols at <https://plos.org/protocols?utm_medium=editorial-email&utm_source=authorletters&utm_campaign=protocols>.

We look forward to receiving your revised manuscript.

Kind regards,

Peter A Newman, Ph.D
Academic Editor
PLOS ONE

Journal Requirements:

When submitting your revision, we need you to address these additional requirements.

1. Please ensure that your manuscript meets PLOS ONE's style requirements, including those for file naming. The PLOS ONE style templates can be found at

<https://journals.plos.org/plosone/s/file?id=wjVg/PLOSOne_formatting_sample_main_body.pdf> and

https://journals.plos.org/plosone/s/file?id=ba62/PLOSOne_formatting_sample_title_authors_affiliations.pdf

 : Thank you, we have checked and followed all instruction as the journal requirements.

2. Please provide additional details regarding participant consent. In the ethics statement in the Methods and online submission information, please ensure that you have specified what type you obtained (for instance, written or verbal, and if verbal, how it was documented and witnessed). If your study included minors, state whether you obtained consent from parents or guardians. If the need for consent was waived by the ethics committee, please include this information.

:   Thank you, we have added information in the section. Informed consent was obtained in written form.

3. When reporting the results of qualitative research, we suggest consulting the COREQ guidelines: <http://intqhc.oxfordjournals.org/content/19/6/349>. In this case, please consider including more information on the number of interviewers, their training and characteristics; and on how participants were selected.

 : Thank you for the suggestion, we have followed the COREQ in whole sections in the paper. We also attach the COREQ within this submission. Please also see the COREQ attached.

4. We note that you have indicated that data from this study are available upon request. PLOS only allows data to be available upon request if there are legal or ethical restrictions on sharing data publicly. For information on unacceptable data access restrictions, please see <http://journals.plos.org/plosone/s/data-availability#loc-unacceptable-data-access-restrictions>.

In your revised cover letter, please address the following prompts;

a) If there are ethical or legal restrictions on sharing a de-identified data set, please explain them in detail (e.g., data contain potentially identifying or sensitive patient information) and who has imposed them (e.g., an ethics committee). Please also provide contact information for a data access committee, ethics committee, or other institutional body to which data requests may be sent.

b) If there are no restrictions, please upload the minimal anonymized data set necessary to replicate your study findings as either Supporting Information files or to a stable, public repository and provide us with the relevant URLs, DOIs, or accession numbers. Please see <http://www.bmj.com/content/340/bmj.c181.long> for guidelines on how to de-identify and prepare clinical data for publication. For a list of acceptable repositories, please see <http://journals.plos.org/plosone/s/data-availability#loc-recommended-repositories>.

We will update your Data Availability statement on your behalf to reflect the information you provide.

 : Data are fully available in appendix section.

[Note: HTML markup is below. Please do not edit.]

Reviewers' comments:

Reviewer's Responses to Questions

**Comments to the Author**

1. Is the manuscript technically sound, and do the data support the conclusions?

The manuscript must describe a technically sound piece of scientific research with data that supports the conclusions. Experiments must have been conducted rigorously, with appropriate controls, replication, and sample sizes. The conclusions must be drawn appropriately based on the data presented.

Reviewer #1: Yes

2. Has the statistical analysis been performed appropriately and rigorously?

Reviewer #1: N/A

3. Have the authors made all data underlying the findings in their manuscript fully available?

The [PLOS Data policy](http://www.plosone.org/static/policies.action#sharing) requires authors to make all data underlying the findings described in their manuscript fully available without restriction, with rare exception (please refer to the Data Availability Statement in the manuscript PDF file). The data should be provided as part of the manuscript or its supporting information, or deposited to a public repository. For example, in addition to summary statistics, the data points behind means, medians and variance measures should be available. If there are restrictions on publicly sharing data—e.g. participant privacy or use of data from a third party—those must be specified.

Reviewer #1: Yes

4. Is the manuscript presented in an intelligible fashion and written in standard English?

PLOS ONE does not copyedit accepted manuscripts, so the language in submitted articles must be clear, correct, and unambiguous. Any typographical or grammatical errors should be corrected at revision, so please note any specific errors here.

Reviewer #1: Yes

5. Review Comments to the Author

Please use the space provided to explain your answers to the questions above. You may also include additional comments for the author, including concerns about dual publication, research ethics, or publication ethics. (Please upload your review as an attachment if it exceeds 20,000 characters)

Reviewer #1: • The authors referred to COVID19 as epidemic, since COVID-19, according to WHO, was declared a pandemic because of the unusually fast rate in which the virus spreads. Pandemic is used when it spreads over significant geographical areas and affects a large percent of the population, while the epidemic is often used broadly to describe any problem that has grown out of control. I would suggest that the authors use the word pandemic instead.

: Thank you, we totally agree with you. It’s improved in whole text including the title.

• Since the informants are hill tribe, the authors explained that ‘However, those who could not speak Thai were helped by village health volunteers who were fluent in both Thai and their local languages’. This process should be further elaborated, the authors should declare how those helpers were trained on research data collection (interview) and ethical considerations.

: Thank you for the great concern. Basically, the helpers just help in translation the message along the interview. During the interview, the interviewer (researchers) were a person on considering the direction of the interview under the question guideline.

: This information had presented during we requested the ethical consideration, but we did not get any comment from the committee.

: However, we agree with you to provide information about the point in the process, please see page 4, lines 130-134.

• Step one: Shock the situation with no prior experience should be changed in the phase that explains the phenomenon. For example, ‘Stage of shock’

: Thank you, we agree with you and have changed accordingly in all points.

• In the conclusion, it is expected to see that the authors can provide some suggestions to the authority and policy makers how to support the hill tribe people to cope with this kind of situation.

: Thank you, we have added recommendations in the conclusion section, please see page 18, lines 537-543.

6. PLOS authors have the option to publish the peer review history of their article ([what does this mean?](https://journals.plos.org/plosone/s/editorial-and-peer-review-process#loc-peer-review-history)). If published, this will include your full peer review and any attached files.


If you choose “no”, your identity will remain anonymous but your review may still be made public.


**Do you want your identity to be public for this peer review?** For information about this choice, including consent withdrawal, please see our [Privacy Policy](https://www.plos.org/privacy-policy).

Reviewer #1: No

Thank you,

TK
