# Supplementary material for: Impacts of and survival adaptations to the COVID-19 pandemic among the hill tribe population of northern Thailand: A qualitative study
Source: PLoS One. 2021 Jun 4;16(6):e0252326. doi: 10.1371/journal.pone.0252326 (PMC8177523; doi:10.1371/journal.pone.0252326)
Supplement: Attachment — Submitted filename: Response to reviewer comment_F.docx [file pone.0252326.s005.docx]

**Responses to reviewer comments**

Thank you for submitting your manuscript to PLOS ONE. After careful consideration, we feel that it has merit but does not fully meet PLOS ONE’s publication criteria as it currently stands. Therefore, we invite you to submit a revised version of the manuscript that addresses the points raised during the review process.

The authors should have the entire manuscript proofread by a professional with proficiency in English before resubmitting):

Line 147: Change to: "Written informed consent was obtained in Thai before starting the interviews."

: Thank you. The whole manuscript has been reedited by American Journal Experts, No A300-3794-F762-55C0-0082.

Line 162: Two SETS OF findings are presented.....  (not "two findings")

: This issue has been addressed, and the whole text has been revised by American Journal Experts, No A300-3794-F762-55C0-0082.

Line 201: Many peope did not RECEIVE support from the government due to their LACK OF Thai citizenship. (And please make sure this is sufficiently explained elsewhere, that some or many "hill tribe" people do not have official Thai citizenship.)

: This issue has been addressed, and the whole text has been revised by American Journal Experts.

: Thank you for the comment; it should be “some” in the context of the hill tribes in Thailand.

Line 286: Many people preferred to REMAIN at their home, while some others TRY TO MAINTAIN THEIR HEALTH BY USING traditional herbs.....

: Thank you so much. This sentence has been revised.

Line 354: Change "fanatical" to FINANCIAL

: Thank you. This word has been changed.

Line 425-6: People BECAME active again in all SECTORS even though this is not the same as BEFORE THE PANDEMIC

: Thank you so much. This issue has been addressed.

Line 537: Delete "An". Essential and accurate information....

: Thank you. This issue has been addressed.

Line 542: Moreover, programS AND interventionS to support AND sustain family FINANCES ARE also recommended.

: Thank you so much. This issue has been addressed.

**Journal Requirements:**

Please review your reference list to ensure that it is complete and correct. If you have cited papers that have been retracted, please include the rationale for doing so in the manuscript text, or remove these references and replace them with relevant current references. Any changes to the reference list should be mentioned in the rebuttal letter that accompanies your revised manuscript. If you need to cite a retracted article, indicate the article’s retracted status in the References list and also include a citation and full reference for the retraction notice.

: Thank you. We have checked all references, and all are correct.

Best,

TK
